# First determination of anticancer, cytotoxic, and in silico ADME evaluation of secondary metabolites of endemic *Astragalus leucothrix* Freyn & Bornm

**DOI:** 10.3906/kim-2104-23

**Published:** 2021-10-05

**Authors:** Ayşe ŞAHİN YAĞLIOĞLU, Duygu GÜNEŞ GÜRBÜZ, Melda DÖLARSLAN, İbrahim DEMİRTAŞ

**Affiliations:** 1 Department of Chemistry and Chemical Process Technology, Technical Sciences Vocational School, Amasya University, Amasya, Turkey; 2 Department Chemistry, Faculty Science, University Çankırı Karatekin, Çankırı, Turkey; 3 Department Biology, Faculty Science, University Çankırı Karatekin, Çankırı, Turkey; 4 Department of Biochemistry, Faculty of Science and Arts, Iğdır University, Iğdır, Turkey

**Keywords:** *Astragalus leucothrix*, anticancer activity, cytotoxic activity, in silico ADME, alfalone, C6 cell

## Abstract

Isolation and characterization of anticancer activity guided secondary metabolites of endemic *Astragalus leucothrix* Freyn& Bornm were aimed. Aerial parts of the plant were extracted by maceration method in the solvent system methanol-chloroform (1 : 1) at room temperature. The obtained crude extract was dissolved in purified water. Then, the extract was partitioned with *n*-hexane, chloroform, ethyl acetate, and *n*-butanol, respectively. Anticancer activity tests of all the fractions were performed against HeLa and C6 cancer cells. The chloroform fraction that has highest anticancer activity was subjected to chromatographic methods such as column chromatography and thin layer chromatography. Pentyl tetratetracontanoate (**1**), alfalone (**2**), 3,6,8-tribromoquinoline (**3**), and 3,6,8-tribromochromenium (**4**) molecules were detected from this plant for the first time. The structure determinations of the isolated molecules were elucidated by methods such as 1D and 2D NMR, HPLC - TOF / MS, and GC - MS analysis. Finally, anticancer and cytotoxic activity tests of the compounds were performed. Literature review showed that 3,6,8-tribromochromenium is a new compound. IC_50_ values of compound **1–2** and compound **3–4** mix were determined to be 4.50 ± 0.10, 2.81 ± 0.00, 4.33 ± 0.00 μM against C6 cell, respectively. The drug likeness properties of **1–4** were obtained by SwissADME. According to Lipinski’s rule of five; **2–4** could be a new potential anticancer agent.

## 1. Introduction

Cancer is a major global public health problem. In addition, the incidence and mortality rates of cancer continue to increase. There will be an estimated 18.1 million new cancer cases and 9.6 million cancer deaths in 2018 [1]. Various systemic treatments such as surgery, chemotherapy, radiotherapy, and hormone therapy are used in cancer treatment [2,3]. Despite these treatment methods, neither a decrease in the number of patients with this disease nor a decrease in the mortality rate is observed [3]. In addition, cancer drugs cause toxicity in normal cells and tissues, causing serious side effects such as vomiting, nausea, hair loss, and resistance development [3–6]. Potential anticancer activities of many medicinal drugs and plant extracts have been investigated in order to avoid these undesirable side effects [3, 7–12]. Therefore, it is extremely important to develop more effective treatments by plants.

*Astragalus* L. (Fabaceae) taxon is one of the largest genera in the world with 2500–3000 taxa [13–16]. In the studies conducted on *Astragalus* taxa in Turkey, it has been reported that there are 425–450 taxa, 201–224 of which are endemic and the rate of endemism varies between 47% and 50% [16,17].

*Astragalus* ssp. includes saponins, flavonoids, and polysaccharides as main classes of compounds [18]. Also, the species contents anthraquinones, alkaloids, amino acids, β-sitosterol, and metallic elements [19]. *Astragalus* species are used as hepatoprotective, antioxidative, immunostimulant, antiviral [20], antidiabetic, cardioprotective, antiinflammatory [19], for the treatment of wounds and leukemia [21, 22], and anticancer [23] at folk medicine. Also, immunomodulatory and anticancer activity of *Astragalus* genus were reported in some studies [24–26]. This pharmacological activity has been determined to be caused by three groups of chemical substances: polyholosites, saponins, and phenolics [20]. To our knowledge, there is no study about the anticancer activity and isolation of endemic *A. leucothrix* Freyn & Bornm. Thus, the main purpose of the research was to investigate the isolation, structural elucidation, biological activities, and in silico ADME evaluation.

## 2. Materials and methods

### 2.1. Chemicals

Fetal bovine serum, penicillin/streptomycin, and Dulbecco’s modified Eagle’s medium-high glucose were purchased from Sigma-Aldrich GmbH. (Germany). Methanol (MeOH), *n*-hexane, chloroform (CHCl_3_), ethyl acetate (EtOAc), and *n*-butanol (BuOH) used in HPLC analysis and extraction were HPLC grade and purchased from Merck. LDH Cell Cytotoxicity Assay (Roche 04 744 926 001, Germany) and BrdU ELISA Assay (Cat. No. 11 647 229 001, Germany) were supplied from Roche.

### 2.2. Plant material

*A. leucothrix* Freyn & Bornm was collected from Yapraklı District (Çankırı, Turkey) in June 2016. The identification of the plant samples was confirmed by botanist Melda DÖLARSLAN. The plants were stored in the Herbarium of Biology Department, Ankara University, Turkey (Herbarium number: ANK 60526).

### 2.3. Extraction and isolation

The dry plant (1 kg) was subjected to maceration method in MeOH-CHCl_3_ (12 L; 1 : 1) to 2 days extraction at room temperature and the procedure was repeated three times. The resulting mixture was then filtered and the solvent was removed in a rotary evaporator. The crude extract was dissolved in purified water (1 L) and was partitioned with *n*-hexane, CHCl_3_, EtOAc, and *n*-BuOH fraction, respectively, and then the solvents were removed to give *n-*hexane (9.42 g), CHCl_3_ (3.73 g), EtOAc (2.40 g), *n*-Butanol (11.79 g), and water (14.85 g) fractions within in the yield of 5.87%, 0.94%, 0.37%, 0.24%, 1.17%, and 1.48%, respectively. CHCl_3_ fraction (3.50 g) was chromatographed by using silica gel column chromatography to give 556 fractions (20 mL, each). The *n*-hexane 1–28 with *n*-hexane-CHCl_3_ (10% ), fraction 29–77 with *n*-hexane-CHCl_3_ (20%); fraction 78–235 with CHCl_3_; fraction 236–299 with acetone-CHCl_3_ (5% ); fraction 300–334 with acetone-CHCl_3_ (10%); fraction 335–365 with acetone-CHCl_3_ (15% ); fraction 366–399 with acetone-CHCl_3_ (50%); fraction 400–423 with acetone-CHCl_3_ (70%); fraction 424–457 with acetone; fractions 458–556 with methanol (MeOH) were eluted. Tubes 114 to 174 were combined by thin layer chromatography. The fractions were subjected to column chromatography with *n*-hexane-CHCl_3_ (50%) again. In the column chromatography was collected 116 fractions. Compound **1** (18 mg), compound **2** (82 mg), and compound **3**–**4** mix (15.6 mg) were isolated from this column.

### 2.4. Determination of anticancer assays

The extract, fractions, and pure compounds were investigated for their anticancer activities against human cervical adenocarcinoma (HeLa) and rat glioma cells (C6) by using BrdU ELISA assays. The tested samples and 5-FU were dissolved in dimethyl sulfoxide (DMSO). Then the stock solution was diluted with Dulbecco’s Modified Eagle Medium (DMEM). DMSO concentration is below 0.1% in stock solutions. The anticancer activity tests and cell culture study were performed according to the literature [27,28]. The results are means ± SD of six values.

### 2.5. Determination of cytotoxic assay

The cytotoxic activities of the test substances were determined according to the manufacturer’s procedure using LDH cell cytotoxicity assay and cytotoxicity % was calculated [29].

### 2.6. Physicochemical and pharmacokinetic properties for computational methods

The SwissADME website was written in HTML, PHP5, and JavaScript, whereas the backend of computation was mainly coded in Python 2.7. The use of additional libraries or software for specific tasks is mentioned in the corresponding paragraph.

The molecule inputted through the sketcher Marvin JS (version 16.4.18, 2016, www.chemaxon.com) are converted into SMILES by JChem Web Services (version 14.9.29, 2013, www.chemaxon.com) installed on one of our servers. This on-the-fly conversion allows seamless paste of SMILES in the input list. The user has the possibility to edit this list as a standard text, e.g., to modify SMILES or add a name to the molecule.^1^ Upon calculation submission by clicking the “Run” button, the SMILES of each molecule is canonicalised by OpenBabel (version 2.3.0, 2012, http://openbabel.org) and processed individually [30]. Drug-likenesses and molecular property predictions of compounds are determined by the programme at http://www.molsoft.com/mprop/mprop.cgi.

### 2.7. Statistical analysis and determination of IC_50_ values

Statistical analyses were used to evaluate anticancer and cytotoxic activity results by one-way ANOVA test. The results are means ± SD of six values. Differences between groups were determined by ANOVA method (p < 0.01). The IC_50_ values were determined using ED50 plus v1.0.

## 3. Results and discussion

### 3.1. Isolation and characterization

**Figure 1:**
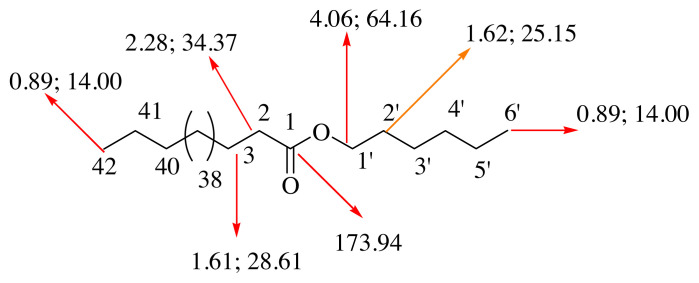
The chemical structure of compound **1** (1st value is proton, 2nd value is carbon values.)

Isolation procedure was started with CHCl_3_ fraction which showed the highest anticancer activity. Three compounds were isolated and identified by spectroscopic methods from the CHCl_3_ fraction. After the TLC analysis, combined fractions 66–81 (274.6 mg) gave the compound **1** (pentyl tetratetracontanoate), which was determined by using 1D and 2D Nuclear Magnetic Resonance (NMR) spectroscopy (Figures S1–S3). When the proton (^1^H) NMR spectrum (600 MHz, CDCl_3_) was examined, two triplet peaks at 0.89 were observed to belong to the terminal methyl proton. The proton of H-1’ was at δ 4.06 and the H-2 proton was resonated as a triplet at δ 2.28. The proton of H-2’ was signalled at δ 1.62 and the proton of H-3 at δ 1.61. Forty-six CH_2_ groups were determined by utilizing the ^1^H NMR spectrum integration values (Figure 1).

When the heteronuclear multiple-bond correlation (HMBC) NMR spectrum of compound **1** was examined, it was determined that the H-8 proton (shown pink) interacts with C-9 and C-7 (carbonyl carbon) carbons. The H-5 proton (shown in red) was shown to correlate with the C-3, C-4, and C-7 carbons (Figure S1). When the carbon (^13^C) NMR spectrum and distortionless enhancement by polarization transfer (DEPT; 150 MHz, CDCl_3_) was examined, the presence of the carbonyl group was determined in δ 173.94 (Figure S2). C-5 carbon was found to be resonance in δ 64.16. The methyl carbons were observed at δ 14.00 (Figures S2 and S3). The compound **1** was isolated from *A. leucothrix* and *Astragalus* ssp. for the first time.

**Figure 2:**
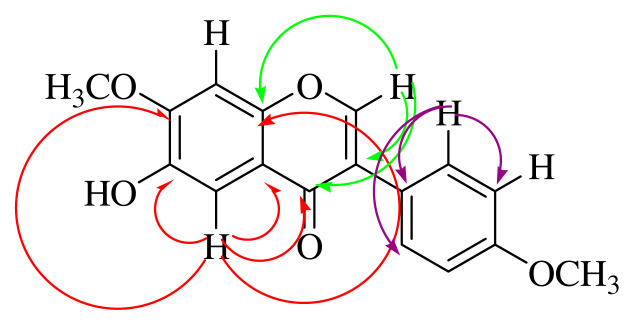
Key HMBC correlation of compound **2**.

After the TLC analysis, fractions 114–174 (82 mg) gave compound **2** (6-hydroxy-7,4’-dimethoxy isoflavone; alfalone; Figure 2) which was determined by the 1D and 2D NMR and HPLC / TOF - MS analysis (Figures S4–S12) [31–33]. Compound **2** was identified as isoflavone (alfalone) that isolated for the first time as a natural product [31]. Alfalone is found in many plants such as *Medicago truncatula* [34], *Trifolium pratense* and *Machaerium isadelphum* [35], and *Machaerium isadelphum* [36]. However, **2** was isolated for the first time from *A. leucothrix* and *Astragalus* ssp. In addition, a large number of isoflavone-type compounds have been isolated from *Astragalus* species. For example, acicerone [37] from *Astragalus cicer*, maackiain from *Astragalus trojanus* [38], diadzen, genisten, and 7-hydroxy-3′,5′-dimethoxyisoflavone from *Astragalus peregrines* [39], 5,5′-dihydroxy-3′-methoxy-isoflavone-7-O-β-d-glucoside, genistin, sissotrin, and 5,4′-dimethoxy-isoflavone-7-O-β-D-glucopyranoside from *Astragalus lycius* Boiss [40].

HPLC/TOF-MS analysis of compound **2** gave [M]^+^ peak at 297.0981 (C_17_H_14_O_5_) (Figures S4 and S5). The singlet peak observed at δ 7.91 in the ^1^H NMR spectrum of compound **2** is characteristic for the H-2 proton in the isoflavone skeleton [39]. The singlet peak in the ^1^H NMR spectrum (Table S1) that were observed in δ 3.83 and 4.01 indicates the methoxy protons linked to the C-4’ and C-7 carbons, respectively, in HMBC spectrum. Protons in the A2X2 system (δ 6.97, dd, 2H, *J* = 2.0, 8.0 Hz; δ 7.50, dd, 2H, *J* = 2.0, 8.0 Hz) are observed to interact with the C-4’ carbon-linked methoxy protons. In the ^1^H NMR spectrum peak at δ 6.27 (1H, s) belongs to the -OH peak due to the D_2_O exchange (Figure S8). Peaks at δ 7.65 and 6.97 (1H) assigned to the H-5 and H-8 protons, respectively (Figure S6). When the ^13^C NMR spectrum of **2** is examined, a signal for the carbonyl group is observed at δ 175.65 and two methoxy peaks at δ 55.46 and 56.66 are observed (Figure S9). When the DEPT of 2 is examined, two CH and five CH signals are observed (Figure S10).

When the HMBC NMR spectrum of compound **2** was examined, it was determined that the H-2 proton (shown green) interacts with C-3, C-4 (carbonyl carbon), and C-9 carbons. The H-5 proton (shown in red) was shown to correlate with the C-4, C-6, C-7, C-9, and C-10 carbons. The H-2’ proton (shown in purple) appears to interact with the C-1’, C-3’, and C-6’ carbons (Figure S7). HSQC spectrum of **2** gave the correlation of peaks; at δ 7.92 with the carbon at δ152.04; δ 7.50 with the carbon at δ 130.58; at δ 7.65 with the carbon at δ 104.96; at δ 6.97 with the carbon at δ 102.74; at δ 6.97 with the carbon at δ 114.38; at δ 4.01 with the carbon at δ 56.66; and at δ 3.83 with the carbon in δ 55.46 (Figure S11). The COSY spectrum of **2** resulted the correlation, H-2’, 6’ at δ 7.50 (2H, *J* = 2.0, 8.0 Hz) with H-3’, 5’ at δ 6.97 (2H, *J* = 2.0, 8.0 Hz) (Figure S12). Thus, the spectral evidence resulted that compound **2** was identified as alfalone which is a known compound [33–35], but it was isolated and characterized first time from this plant.

The fractions 114–174 (15.6 mg) were seen as a pure compound in TLC, HPLC/TOF-MS and GC-MS analyses (Figures S13 and S14). However, 3,6,8-tribromoquinoline (**3**) and 3,6,8-tribromochromenium (**4**) mix were obtained which were determined by 1D and 2D NMR analysis (Figures S15–S21, Table 1). When the spectra at Figure S16 and S17 are examined; two peaks were observed to overlap at 152.47, 136.64, 130.72, 128.76, 121.22, and 119.31 ppm. In addition, when Figure S17 was examined; another peak was detected at 174.21 ppm. When Kar et al., 2021 was examined, it was determined that the C2 carbon in benzopyrylium ions had a resonance between 169.20 and 179.60 ppm. Also, carbon C9 resonates between 155.90 and 167.00 ppm depending on the substituent at C6 and/or C8 [41]. However, all peaks except carbons C2 and C9 are compatible with compound **3** (3,6,8-tribromoquinoline)[42–43]. Considering the peaks, the structure was determined to be compound **4** (3,6,8-tribromobenzopyryllium, Table 1). In addition, when the spectrums at Figure S16 are examined; nine peaks were determined 152.47, 142.36, 136.64, 136.31, 130.72, 128.76, 126.03, 121.22, and 119.31 ppm. The peaks are compatible with compound **3** [42–43].

The quinoline skeleton is found in a variety of natural compounds and synthetic derivatives. It has many biological activities such as antimalarial, antibacterial, antifungal, anthelmintic, cardiotonic, anticonvulsant, anti-inflammatory, and analgesic activity [44]. However, quinolone derivatives were isolated from many plants such as *Ephedra pachyclada* ssp. *sinaica* [45], *Haplophyllum foliosum*, *Haplophyllum pedicellatum* [46], *Solidago canadensis* [47], *Eremophila microtheca* [48], *Lunasia amara* [49], and *Pitaviaster haplophyllus* [50].

In addition, the first naturally occurring bromo-quinoline alkaloid was isolated from the marine bryozoan *Flustra foliacea* (L.) [51].

**Figure 3:**
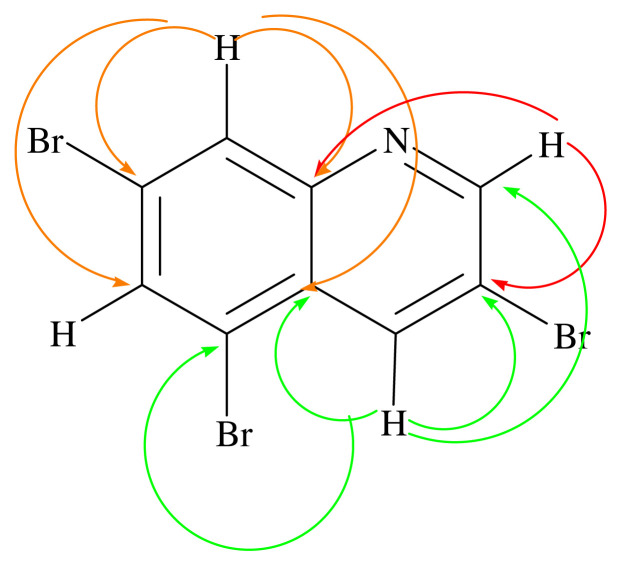
Key HMBC correlation of compound **3–4** mix.

In ^1^H NMR spectrum (600 MHz, CDCl_3_, Figure S15, Table 1) of compound **3**–**4** mix, H-2 proton peak at δ 9.00 (2H, s), H-4 proton at δ 8.24 (2H, s), H-6 proton at δ 7.88 (2H, s), and H-8 proton at δ 8.15 (2H, s) were determined [42]. In ^13^C NMR spectrum and HPLC-TOF/MS analysis of compound **3**–**4** mix, compound **3** was observed as [C_9_H_4_Br_3_N]^+^ and [M]^+^ peak m/z at 367.8055 (Figure S20). At the same time, compound **4** was detected as [C_9_H_4_Br_3_O]^+^, [M]^+^ peak m/z at 365.8074 (Figure S20). The nitrogen rule in mass spectrometry states that (organic) molecules containing no or an even number of nitrogen atoms will have even masses, and molecules containing an odd number of nitrogen atoms will have odd masses [52]. Thus, the fact that the molecular ion peak in the mass spectrum is a single number supports the presence of nitrogen atom in the structure. In addition, when the mass spectrum was examined, three bromine atoms were observed in both molecules (Figure S20)[53]. ^13^C and DEPT NMR spectra of compound **3** (150 MHz, CDCl_3_) gave nine signals at δ 152.47, 119.24, 136.64, 121.22, 128.76, 126.03, 136.31, 142.36, and 130.72, which were assigned to carbons C-2, C-3, C-4, C-5, C-6, C-7, C-8, C-9, and C-10, respectively [42]. In the HMBC spectrum (600 MHz, CDCl_3_) in Figure 3, the H-2 proton (shown in red) was found to interact with the C-3, C-4, and C-9 carbons. The H-4 proton (shown in green) correlates with the C-2, C-3, C-5, and C-10 carbons. The H-8 proton (shown in pink) interacts with C-6, C-7, C-9, and C-10 carbons (Figures 3 and S21). When the HSQC spectrum (600 MHz, CDCl_3_) in Figure S19 was examined, the proton in δ 9.00 ppm with carbon at δ152.47 ppm, the proton in δ 8.24 ppm with carbon in δ136.64 ppm, the proton in δ 8.15 ppm with the carbon in δ136.31 ppm, and the proton in δ 7.88 ppm with the carbon in the carbon δ128.66 ppm were seen to be correlated. The interactions overlap with compound **3**. Bromine ranks 44th among the elements found in the earth’s crust. There are many organobromine compounds synthesized by living organisms or formed as a result of natural abiotic processes [54]. Because of their similar physical and chemical properties, bromides are commonly found in the environment together with sodium chloride in smaller amounts. Br has been shown to be a new and important trace element for humans and animals [55]. Although various plant species can accumulate high concentrations of Br, to our knowledge, their role in plants has not been established [56]. In marine plants (for example, *Bonnemaisonia hamifera*, *Laurencia* species), marine animals (for example, sponges, bryozoans, corals), mammals (for example, cat and rat), abiogenic sources, plants (for example, rapeseed, mustard, cabbage, Chinese cabbage, broccoli, pak-choi, alyssum, wild mustard, turnip, radish), fungi and lichen, bacteria (for example, *Bacillus subtilis, Chromobacterium* species), and insects are naturally found organobromine compounds [57]. However, the compound **3** and **4** were isolated from *A. leucothrix* and *Astragalus* ssp. for the first time.

### 3.2. Anticancer activity

**Figure 4:**
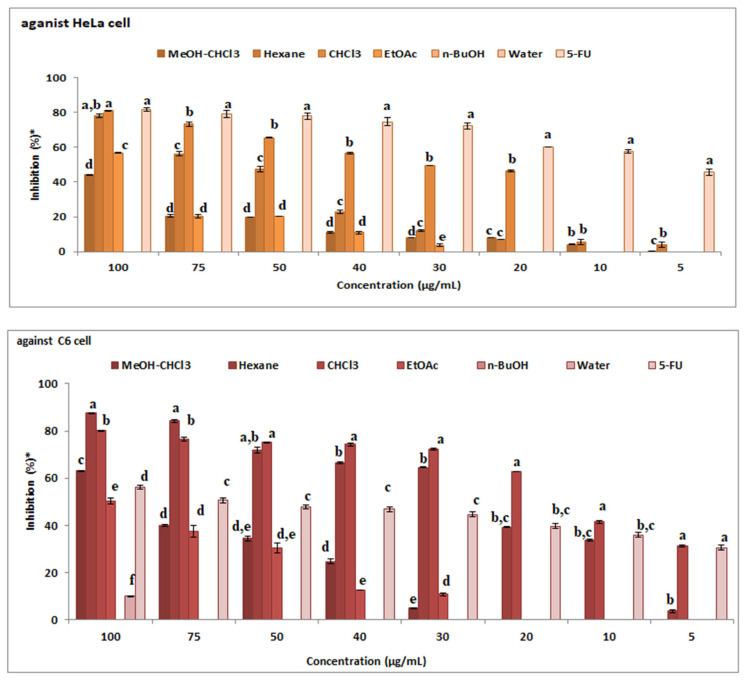
The anticancer activity of extracts against C6 and HeLa cell (* tests repeated three times and twice).

The anticancer activities of MeOH: CHCl_3_ extract, *n*-hexane, CHCl_3_, EtOAc, *n-*Butanol and water fractions and 5-FU that were used as a standard against C6 and HeLa cell were investigated (Figure 4). As a result of the tests performed, an increase in the activity of all extracts due to dose increase was observed. Activity at 100 μg/mL concentration against HeLa cell (Figure 4): CHCl_3_ fraction > 5-FU > *n*-hexane fraction > EtOAc fraction > MeOH: CHCl_3_ extract > *n*-BuOH fraction > water fraction. When cancer activity results were examined in both cells, the most active fraction was found to be chloroform fraction. The highest activity against C6 cells was observed in *n*-hexane and chloroform fractions (Figure 4). Activity at 100 μg/mL concentration against C6 cell: *n*-hexane fraction > CHCl_3_ fraction > MeOH: CHCl_3_ extract > 5-FU > EtOAc fraction > water fraction > *n*-BuOH fraction.

In cytotoxicity studies on *Astragalus chrysochlorus* extracts, the highest effect was observed in chloroform extract. This extract was followed by ethyl acetate, ethanol, *n*-hexane, and aqueous ethanol extracts [58]. Similarly, in our study, the highest effect was observed in chloroform extract. This extract was followed by *n*-hexane extract. We performed GC-MS analyses of *n*-hexane and chloroform extracts of *A. leucothrix* (Table 2). In the *n*-hexane extract, palmitic acid, linolenic acid, and linoleic acid were main components. Palmitic acid, linolenic acid, and behenic acid were major compounds in chloroform extracts. PUFA fatty acids are used in chemotherapy. They also increase the effectiveness of chemotherapeutic drugs and may reduce chemotherapy or cancer side effects. Linolenic acid in the chloroform extract inhibited various cancer cells such as GOTO, SK-N-DZ, DU145, A-549, PC-3, 36B10 cells [59]. Also, palmitic acid has anticancer activity against human leukemic cell line (MOLT-4), colon 26 murine tumour cells, and human breast cancer (MCF-7). It is also known that the crude extracts are more effective than their pure compounds for pharmacologically. This effect is thought to be due to the synergistic effect of many molecules in the extracts [60].

**Figure 5:**
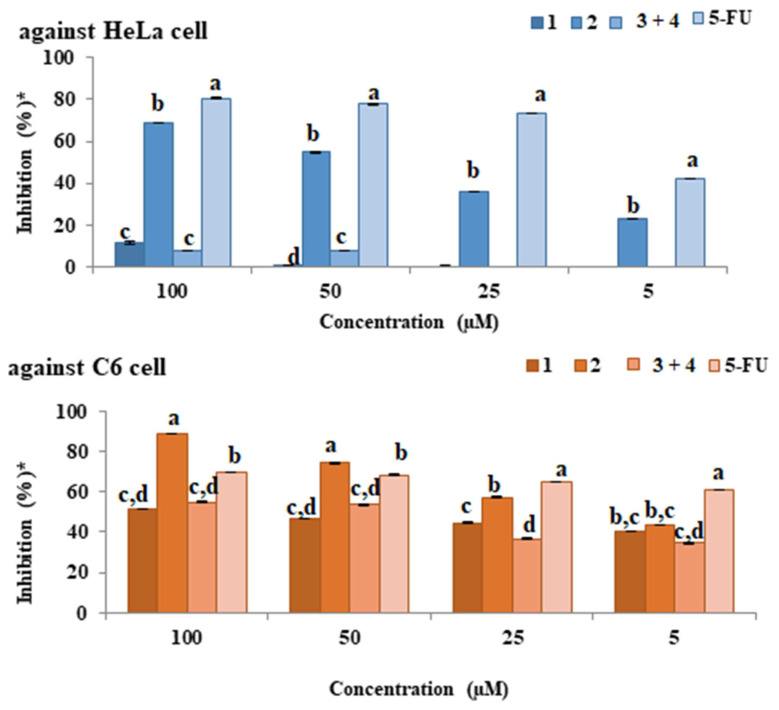
The anticancer activity of the compounds against C6 and HeLa cell (* tests repeated three times and twice).

The anticancer activities of the isolated compound **1–2**, compound **3–4** mix, and 5-FU used as standard were examined against the HeLa as a result of the tests (Figure 5), an increase was observed in all molecules (except compound **3–4** mix) due to dose increase. Among the isolated molecules, the highest activity against the HeLa cell was observed in the compound **2**.

Activity at a concentration of 100 μM is as follows: 5-FU > compound **2** > compound **1** > compound **3–4** mix. As a result of anticancer activity tests of compound **1–2**, compound **3–4** mix, and 5-FU against C6 cells (Figure 5), an increase was observed in all molecules due to dose increase. The highest activity against the C6 cell between the isolated molecules and the 5-FU was observed in the compound **2**. Cell selective activity against C6 cells was observed in all isolated molecules. The activity at 100 μM concentration is compound **2** > 5-FU > compound **3–4** mix > compound **1**. The anticancer activities of the isolated compound **1–2**, compound **3–4** mix, and 5-FU used as standard were examined against the HeLa and C6 cells. The IC_50_ values of these compounds are given in Table 2.

As in this study, the isolated compounds and extracts from *Astragalus* species such as *Astragalus tribuloides* [60], *Astragalus hamosus* [60–62], *Astragalus membranaceus* [25, 63–64], *Astragalus ovinus* [65], *Astragalus vogelii* [66], *Astragalus complanatus* [67] have anticancer activity.

### 3.3. Cytotoxic activity

C6 cells were used to determine cytotoxic activity. 100 μg/mL concentration, which is the highest dose used in the anticancer activity tests, was also studied during the experiment. 5-FU was used as a positive control. Test results were given in Table 1. The cytotoxicity values of the samples are relatively small (except compound **2**) compared to 5-FU. Especially compound **3–4** mix is less toxic than 5-FU.

### 3.4. Drug likeness properties

The number of hydrogen bond acceptors (n-ON) and donors (n-OHNH) are within the Lipinski’s rules, n-ON < 10 and n-OHNH < 5. The calculated log P must be smaller than 5. In our study, the log P values of compound **2–4** were smaller than 5. The molecular weight of the compounds is in the range of 298.29 g/mol and 719.30 g/mol, respectively. The blood-brain barrier (BBB) score: 6-High, 0-Low [68]. The BBB score of compound **1–4** ranges from 3.23 to 4.28. Compound **1–4** can cross the BBB. Synthetic accessibility score of the compounds are from 1 (very easy) to 10 (very difficult). Synthetic accessibility of all the compounds is in the range of 1.79 and 6.77. Topological polar surface area (TPSA) must be <70 Å^2^. TPSA values of all the compounds were smaller than 70 Å^2^ (Tables 3 and 4).

**Figure 6:**
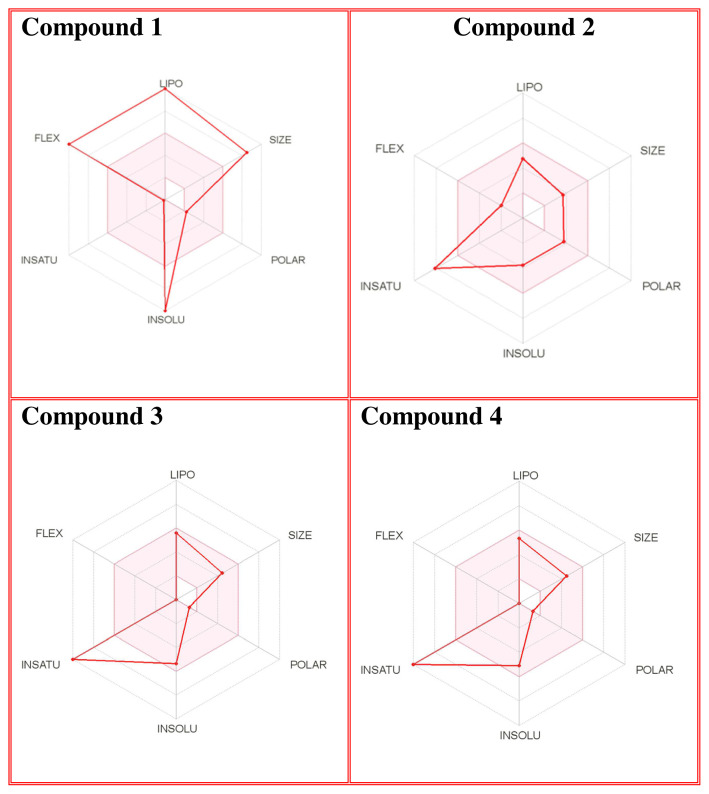
The bioavailability radar of the compounds **1–4**.

The solubility (log S) scale value ranges between −10 (insoluble), −6 (poorly soluble), −4 (soluble), −2 (very soluble), and 0 (highly soluble). The solubility values of compound **1–4** were −16.56, −3.77, −5.36, and 5.10, which respectively correspond to insoluble, soluble, moderately soluble, and moderately soluble. The more negative the skin permeation (log Kp) the less the skin-permeant the molecule. For example, Diclofenac is a good topic antiinflammatory with a predicted log Kp of −4.96 (cm/s), while Ouabain has little chance to cross skin with a predicted log Kp of −10.94 (cm/s). The log Kp values of compound **1–4** were 6.62, −6.15, −5.51, and −5.83 cm/s, respectively. The Kp values showed that compound **2–4** was good in skin permeability (Tables 3 and 4). According to Lipinski’s rule of five, compound **2–4** could be a new potential anticancer agent according to calculated data (Tables 3 and 4) [69]. The pink area represents the optimal range for each property (lipophilicity: LOGP between −0.7 and +5.0, size: MW between 150 and 500 g/mol, polarity: TPSA between 20 and 130 Å^2^, solubility: log S not higher than 6, saturation: fraction of carbons in the sp3 hybridization not less than 0.25, and flexibility: no more than 9 rotatable bonds. In this example, compound **2–4** are predicted orally bioavailable, because of being flexible, polar, and small size. Bioavailability radar of compound **1–4** is demonstrated in Figure 6.

## Suplemantary materials

Figure S1 ^1^H NMR spectrum and HMBC correclations of compound **1** (600 MHz, CDCl_3_)

Figure S2 ^13^C NMR spectrum of compound **1** (150 MHz, CDCl_3_)

Figure S3 DEPT NMR spectrum of compound **1** (150 MHz, CDCl_3_)

Figure S4 HPLC/TOF-MS chromatogram of compound **2**

Figure S5 Mass spectrum of compound **2**

Figure S6 ^1^H NMR spectrum of compound **2** (600 MHz, CDCl_3_)

Figure S7 HMBC spectrum of compound **2** (600 MHz, CDCl_3_)

Figure S8 ^1^H NMR spectrum of compound **2** taken with D_2_O (600 MHz, CDCl_3_)

Figure S9 ^13^C NMR spectrum of compound **2** (150 MHz, CDCl_3_)

Figure S10 DEPT NMR spectrum of compound **2** (150 MHz, CDCl_3_)

Figure S11 HSQC NMR spectrum of compound **2** (600 MHz, CDCl_3_)

Figure S12 COSY NMR spectrum of compound **2** (600 MHz, CDCl_3_)

Figure S13 HPLC/TOF-MS chromatogram of compound **3**–**4** mix

Figure S14 GC-MS chromatogram of compound **3**–**4** mix

Figure S15 ^1^H NMR spectrum of compound **3**–**4** mix (600 MHz, CDCl_3_)

Figure S16 ^13^C NMR spectrum of compound **3**–**4** mix (150 MHz, CDCl_3_)

Figure S17 DEPT NMR spectrum of compound **3**–**4** mix (150 MHz, CDCl_3_)

Figure S18 COSY spectrum of compound **3**–**4** mix (600 MHz, CDCl_3_)

Figure S19 HSQC spectrum of compound **3**–**4** mix (600 MHz, CDCl_3_)

Figure S20 The mass spectra and structure of compound **3**–**4** mix

Figure S21 HMBC NMR spectrum and correlations of compound **3**–**4** mix (600 MHz, CDCl_3_)

**Table S1:** ^1^H, ^13^C NMR, and DEPT data of compound **2**

Position	^1^H	^13^C	DEPT
Compound **2**			
2	7.92 (1H, *s*)	152.04	CH
3	-	124.17	C
4	-	175.65	C
5	7.65 (1H, *s*)	104.96	CH
6	-	145.53	C
7	-	152.45	C
8	6.97 (1H, *s*)	102.47	CH
8a	-	151.15	C
4a	-	117.93	C
1′	-	124.31	C
2′	7.50 (2H, *dt*, *J*=2.0, 8.0 Hz)	130.58	CH
3′	6.97 (2H, *dt*, *J*=2.0, 8.0 Hz)	114.38	CH
4′	-	159.49	C
5′	6.97 (2H, *dt, J*=2.0, 8.0 Hz)	114.38	CH
6′	7.50 (2H, *dt, J*=2.0, 8.0 Hz)	130.58	CH
OMe	3.83 (3H, *s*)	55.46	CH _3_
	4.01 (3H, *s*)	56.66	CH _3_
OH	6.27 (1H, *s*)	-	-

**Figure:**
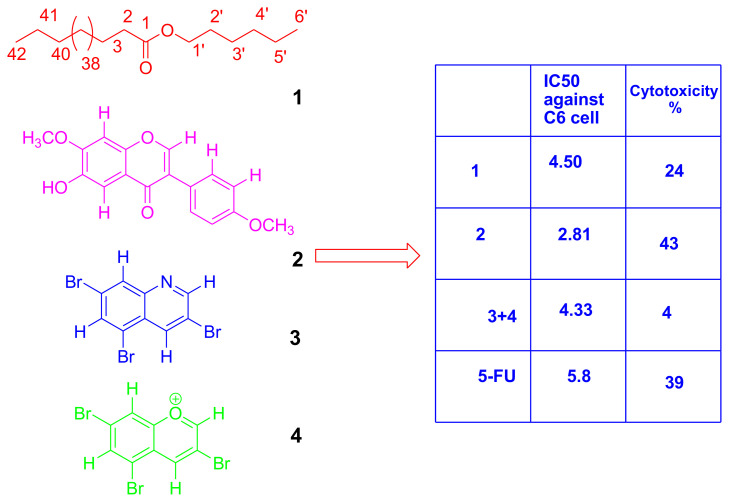

**Highlights**

1. Four compound were isolated firstly from *Astragalus leucothrix*.
2. Two tribromo compound was identified for the first time as a natural product.
3. According to Lipinski’s rule of five; **2**–**4** could be a new potential anticancer agent.

## Figures and Tables

**Table 1:** NMR data of compounds **3**–**4** (CDCl_3_, 600 and 150 MHz); IC_50_ values and cytotoxicity (%) of the compounds.

Compound **3** (3,6,8-tribromoquinoline)					
	Detected	Literature (ppm) [41–43]	Detected (ppm)	Literature (ppm) [41–43]	Detected/ literature
2	9.00 (1H, *bs*)	8.99 (1H,d*)	152.47	153.42	CH/ CH
3	-	-	119.24	120.06	C
4	8.24 (1H, *bs*)	8.20 (1H, d*)	136.64	137.51	CH/ CH
5	-	-	121.22	121.97	C
6	7.88 (1H,*bs*)	7.86 (1H,d*)	128.76	129.57	CH/ CH
7	-	-	126.03	126.77	C
8	8.15 (1H, *bs*)	8.14 (1H, d*)	136.31	137.15	CH/ CH
9	-	-	142.36	143.18	C
10	-	-	130.72	131.50	C
Compound **4** (3,6,8-tribromobenzopyrylium)					
	Detected (ppm)	Literature [41–43]	Detected (ppm)	Literature (ppm) [41–43]	Detected/ literature
2	9.00 (1H, *bs*)	8.99 (1H,d*)	174.21	169.20–179.60 ^41^	CH/ CH
3	-	-	119.31	120.06	C
4	8.24 (1H, *bs*)	8.20 (1H, d*)	136.64	137.51	CH/ CH
5	-	-	121.27	121.97	C
6	7.88 (1H,*bs*)	7.86 (1H,d*)	128.76	129.57	CH/ CH
7	-	-	126.03	126.77	C
8	8.15 (1H, *bs*)	8.14 (1H, d*)	136.31	137.15	CH/ CH
9	-	-	152.47	155.90–167.00 ^41^	C
10	-	-	130.72	131.50	C
		**HeLa cell**	**C6 cell**		**Cytotoxicity (%)**
Compound 1		72.35 ± 0.51^a^	4.50 ± 0.10^a^		24.25 ± 0.01^c^
Compound 2		22.07 ± 0.21^b^	2.81 ± 0.00^b^		43.02 ± 0.02^a^
Compound **3**+**4**		73.22 ± 0.25^a^	4.33 ± 0.00^a^		4.05 ± 0.01^d^
5-FU		16.32 ± 0.11^c^	5.8 ± 0.10^c^		39.02 ± 0.03^b^

* Signals specified as doublets appear as broad singlets in the article [42].

**Table 2:** GC-MS analysis results of the *n*-hexane and chloroform extracts of *A. leucothrix*.

No	RT	Isomer	Compound name	Area%	
				*n*-Hexane	CHCl_3_
Saturated Fatty Acids (SFAs)					
**1**	19.834	C_12_:0	Lauric acid	-	0.86
**2**	22.912	C_14_:0	Myristic acid	1.36	1.04
**3**	27.650	C_16_:0	Palmitic acid	19.72	18.30
**4**	32.583	C_18_:0	Stearic acid	4.41	5.02
**5**	34.854	C_20_:0	Arachidic acid	3.14	3.27
**6**	37.320	C_22_:0	Behenic acid	2.56	11.27
**7**	45.577	C_23_:0	Tricosylic acid	-	2.50
**8**	40.622	C_24_:0	Lignoceric acid	-	4.89
Subtotal				31,19	57,15
Polyunsaturated Fatty Acids (PUFAs)					
**9**	32.193	C_18_:2	Linoleic acid	11.41	4.81
**10**	32.302	C_18_:3	Linolenic acid	28.32	13.30
Subtotal				39.73	18.11
Other Components					
**11**	32.445		3,7,11,15-Tetramethyl-2-hexadecen-1-ol	2.42	1.53
**12**	36.834		Behenic alcohol	-	1.76
**13**	39.998		Heptacosane	2.39	2.78
**14**	44.616		Nonacosane	10.31	8.79
**15**	51.992		Hentriacontane	2.90	9.85
**16**	64.214		*γ*-Sitosterol	11.06	-
**17**	53.336		Octacosanoic acid, methyl ester	-	0.36
Subtotal				26.18	25.07
General Total				97.1	100.3

**Table 3:** Physicochemical properties, lipophilicity, solubility, pharmacokinetics, drug likeness, and medicinal chemistry of compound **1**–**4** predicted using Swiss ADME.

No	Physicochemical properties	Lipophilicity	Water solubility	Pharmacokinetics	Drug likeness	Medicinal chemistry
**1**	Formula: C49H98O2 Moleculer weight: 719.30 g/ mol Num. heavy atoms: 51 Num. arom.heavy atoms: 0 Fraction Csp3:0.98 Num. rotatable bonds: 47 Num. H-bond acceptors: 2 Num. H-bond donors: 0 Molar Refractivity: 238.94 TPSA: 26.30 Å^2^	Log P_o/W_ (iLOGP): 11.92 Log P_o/W_ (XLOGP3): 24.38 Log P_o/W_ (WLOGP): 18.12 Log P_o/W_ (MLOGP): 10.48 Log P_o/W_ (SILICOS-IT): 19.98 Consensus Log P_o/W_: 16.98	Log *S* (ESOL): −16.56 Solubility: 1.99e-14 mg/ml ; 2.77e-17 mol/l Class: Insoluble Log *S* (Ali): −25.40 Solubility: 2.85e-23 mg/ml ; 3.96e-26 mol/l Class: Insoluble Log *S*(SILICOS-IT): −18.49 Solubility: 2.33e-16 mg/ml ; 3.24e-19 mol/l Class: Insoluble	GI absorption:Low P-gp substrate: Yes CYP1A2 inhibitor: No CYP2C19 inhibitor:No CYP2C9 inhibitor:No CYP2D6 inhibitor:No CYP3A4 inhibitor:No Log Kp (skin permeation): 6.62 cm/s	Lipinski: No; 2 violations: MW>500, MLOGP>4.15 Ghose: No; 4 violations: MW>480, WLOGP>5.6, MR>130, #atoms>70 Veber: No; 1 violation: Rotors>10 Egan: No; 1 violation: WLOGP>5.88 Muegge: No; 3 violations: MW>600, XLOGP3>5, Rotors>15 Bioavailability Score: 0.17	PAINS: = 0 alert Brenk: 0 alert Leadlikeness: No; 3 violations: MW>350, Rotors>7, XLOGP3>3.5 Synthetic accessibility: 6.77
**2**	Formula: C17H14O5 Moleculer weight: 298.29 g/ mol Num. heavy atoms: 22 Num. arom.heavy atoms: 16 Fraction Csp3:0.12 Num. rotatable bonds: 3 Num. H-bond acceptors: 5 Num. H-bond donors: 1 Molar Refractivity: 82.93 TPSA: 68.90 Å^2^	Log Po/W (iLOGP): 2.95 Log Po/W (XLOGP3): 2.77 Log Po/W (WLOGP): 3.18 Log Po/W (MLOGP): 1.01 Log Po/W (SILICOS-IT): 3.55 Consensus Log Po/W: 2.69	Log S (ESOL): −3.77 Solubility: 5.01e-02 mg/ml ; 1.68e-04 mol/l Class: Soluble Log S (Ali): −3.87 Solubility: 4.00e-02 mg/ml ; 1.34e-04 mol/l Class: Soluble Log S(SILICOS-IT): −5.80 Solubility: 4.74e-04 mg/ml ; 1.59e-06 mol/l Class: Moderately soluble	GI absorption: High P-gp substrate:No CYP1A2 inhibitor: Yes CYP2C19 inhibitor: No CYP2C9 inhibitor: Yes CYP2D6 inhibitor: Yes CYP3A4 inhibitor: Yes Log Kp (skin permeation): −6.15 cm/s	Lipinski: Yes; 0 violation Ghose: Yes Veber: Yes Egan: Yes Muegge: Yes Bioavailability Score: 0.55	PAINS: 0 alert Brenk: 0 alert Leadlikeness: Yes Synthetic accessibility: 3.04
**3**	Formula: C9H4Br3N Moleculer weight: 365.85 g/ mol Num. heavy atoms: 13 Num. arom.heavy atoms: 10 Fraction Csp3:0.00 Num. rotatable bonds: 0 Num. H-bond acceptors: 1 Num. H-bond donors: 0 Molar Refractivity: 64.84 TPSA: 12.89 Å^2^	Log P_o/W_ (iLOGP): 2.79 Log P_o/W_ (XLOGP3): 4.26 Log P_o/W_ (WLOGP): 4.52 Log P_o/W_ (MLOGP): 3.93 Log P_o/W_ (SILICOS-IT): 4.44 Consensus Log P_o/W_: 3.99	Log *S* (ESOL): −5.36 Solubility: 1.59e-03 mg/ml; 4.35e-06 mol/l Class: Moderately soluble Log *S* (Ali): −4.24 Solubility: 2.09e-02 mg/ml; 5.72e-05 mol/l Class: Moderately soluble Log *S*(SILICOS-IT): −6.21 Solubility: 2.28e-04 mg/ml ; 6.22e-07 mol/l Class: Poorly soluble	GI absorption: High P-gp substrate: No CYP1A2 inhibitor:Yes CYP2C19 inhibitor: Yes CYP2C9 inhibitor: Yes CYP2D6 inhibitor:No CYP3A4 inhibitor:No Log Kp (skin permeation): −5.51 cm/s	Lipinski: Yes; 0 violation Ghose: No; 1 violation: #atoms<20 Veber: Yes Egan: Yes Muegge: No; 1 violation: Heteroatoms <2 Bioavailability Score: 0.55	PAINS: 0 alert Brenk: 0 alert: Leadlikeness: No; 2 violations: MW>350, XLOGP3>3.5 Synthetic accessibility: 1.79
**4**	Formula: C9H4Br3O Moleculer weight:367.84 g/ mol Num. heavy atoms: 13 Num. arom.heavy atoms: 10 Fraction Csp3: 0.00 Num. rotatable bonds: 0 Num. H-bond acceptors: 1 Num. H-bond donors: 0 Molar Refractivity: 63.72 TPSA: 13.14 Å^2^	Log P_o/W_ (iLOGP): −2.05 Log P_o/W_ (XLOGP3): 3.82 Log P_o/W_ (WLOGP): 5.00 Log P_o/W_ (MLOGP): 3.93 Log P_o/W_ (SILICOS-IT): 3.16 Consensus Log P_o/W_: 2.77	Log *S* (ESOL): −5.10 Solubility2.95e-03 mg/ml ; 8.01e-06 mol/l Class: Moderately soluble Log *S* (Ali): −3.79 Solubility: 5.95e-02 mg/ml ; 1.62e-04 mol/l Class: Soluble Log *S*(SILICOS-IT): −5.30 Solubility: 1.83e-03 mg/ml ; 4.98e-06 mol/l Class: Moderately soluble	GI absorption: High P-gp substrate:Yes CYP1A2 inhibitor:No CYP2C19 inhibitor:No CYP2C9 inhibitor:No CYP2D6 inhibitor:No CYP3A4 inhibitor:No Log Kp (skin permeation): −5.83 cm/s	Lipinski: Yes; 0 violation Ghose: No; 1 violation: #atoms<20 Veber: Yes Egan: Yes Muegge: No; 1 violation: Heteroatoms <2 Bioavailability Score: 0.55	PAINS: 0 alert Brenk: 1 alert: charged_ oxygen_sulfur Leadlikeness: No; 2 violations: MW>350, XLOGP3>3.5 Synthetic accessibility: 2.81

**Table 4:** SMILES, Lipinski’s rule of five and drug likeness of compound **1–4** predicted using molsoft programme.

No	SMILES	Moleculer properties	Drug likeness
**1**	CCCCCCCCCCCCCCC CCCCCCCCCCCCCCC CCCCCCCCCCCCCC( =O)OCCCCC	Moleculer formula: C49 H98 O2 Moleculer weight: 718.76 (> 500) Number of HBA: 2 Number of HBD: 0 MolLogP: 23.34 (> 5) MolLogS: −5.79 (in Log(moles/L)) 1.16 (in mg/L) MolPSA: 20.96 A^2^ MolVol: 901.53 A^3^ Number of stereo centers: 0 BBB Score: 3.23	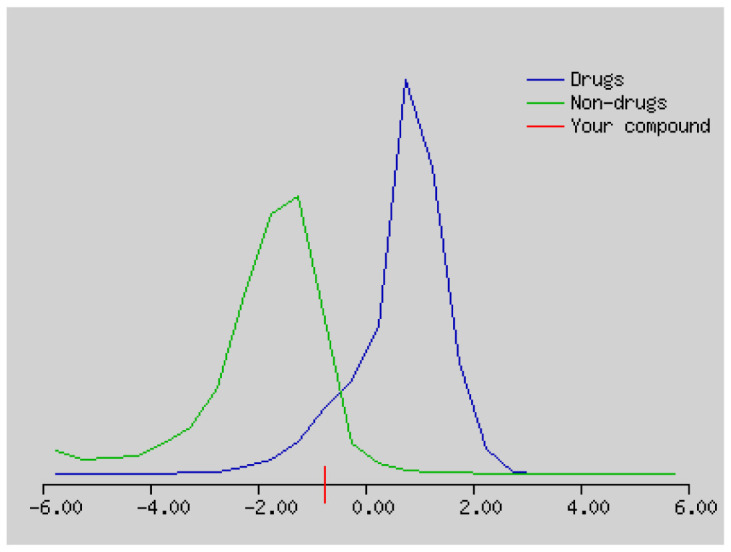 Drug-likeness model score: −0.76
**2**	[H]C1=C(C(=O) C2=C(O1) C([H])=C(OC) C(O)=C2[H])C1=C([H]) C([H])=C(OC) C([H])=C1[H]	Moleculer formula:C17 H14 O5 Moleculer weight: 298.08 Number of HBA: 5 Number of HBD: 1 MolLogP: 2.54 MolLogS: −2.91 (in Log(moles/L)) 370.00 (in mg/L) MolPSA: 52.58 A^2^ MolVol: 304.52 A^3^ Number of stereo centers: 0 BBB Score: 3.93	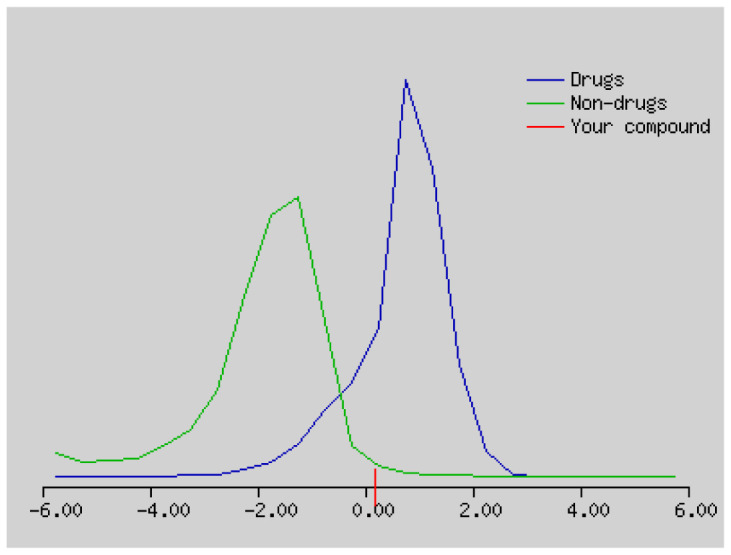 Drug-likeness model score: 0.17
**3**	BrC1=CC2=C(C=C(Br) C=C2Br)N=C1	Moleculer formula:C9 H4 Br3 N Moleculer weight: 362.79 Number of HBA: 1 Number of HBD: 0 MolLogP: 4.91 MolLogS: −5.04 (in Log(moles/L)) 3.34 (in mg/L) MolPSA: 9.86 A^2^ MolVol: 186.91 A^3^ Number of stereo centers: 0 BBB Score: 4.28	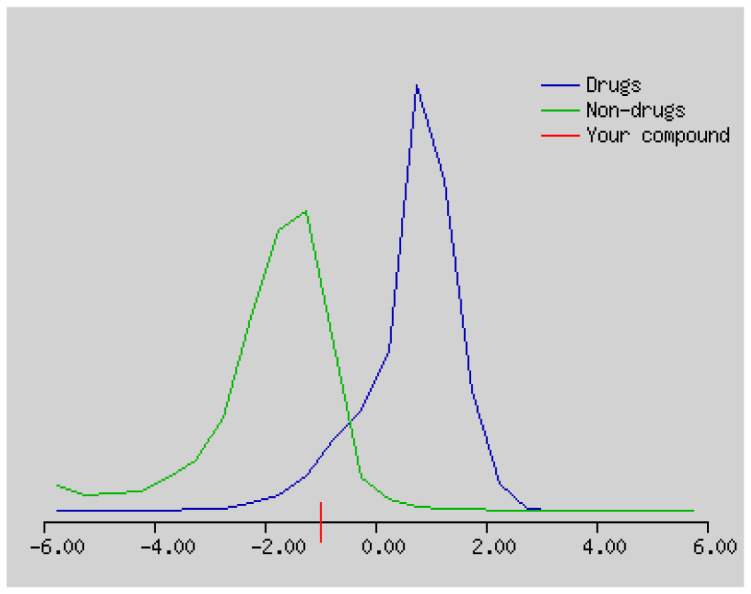 Drug-likeness model score: −0.97
**4**	BrC1=CC2=C(C=C(Br) C=C2Br)[O+]=C1	Moleculer formula:C9 H4 Br3 O Moleculer weight: 364.78 Number of HBA: 1 Number of HBD: 0 MolLogP: 4.52 MolLogS: −4.43 (in Log(moles/L)) 13.44 (in mg/L) MolPSA:5.95 A^2^ MolVol: 201.83 A^3^ Number of stereo centers: 0 BBB Score: 4.15	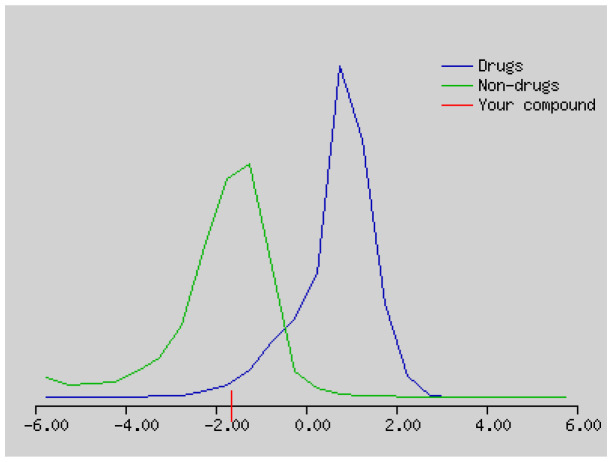 Drug-likeness model score: −1.64

## Footnotes

1 www.nature.com/scientificreports/SCientifiC REpOrtS | 7:42717 | DOI: 10.1038/srep42717
