# Supplementary figures and images for: First determination of anticancer, cytotoxic, and in silico ADME evaluation of secondary metabolites of endemic Astragalus leucothrix Freyn & Bornm
Source: Turk J Chem. 2021 Oct 5;46(1):169–83. doi: 10.3906/kim-2104-23 (PMC10734755; doi:10.3906/kim-2104-23)

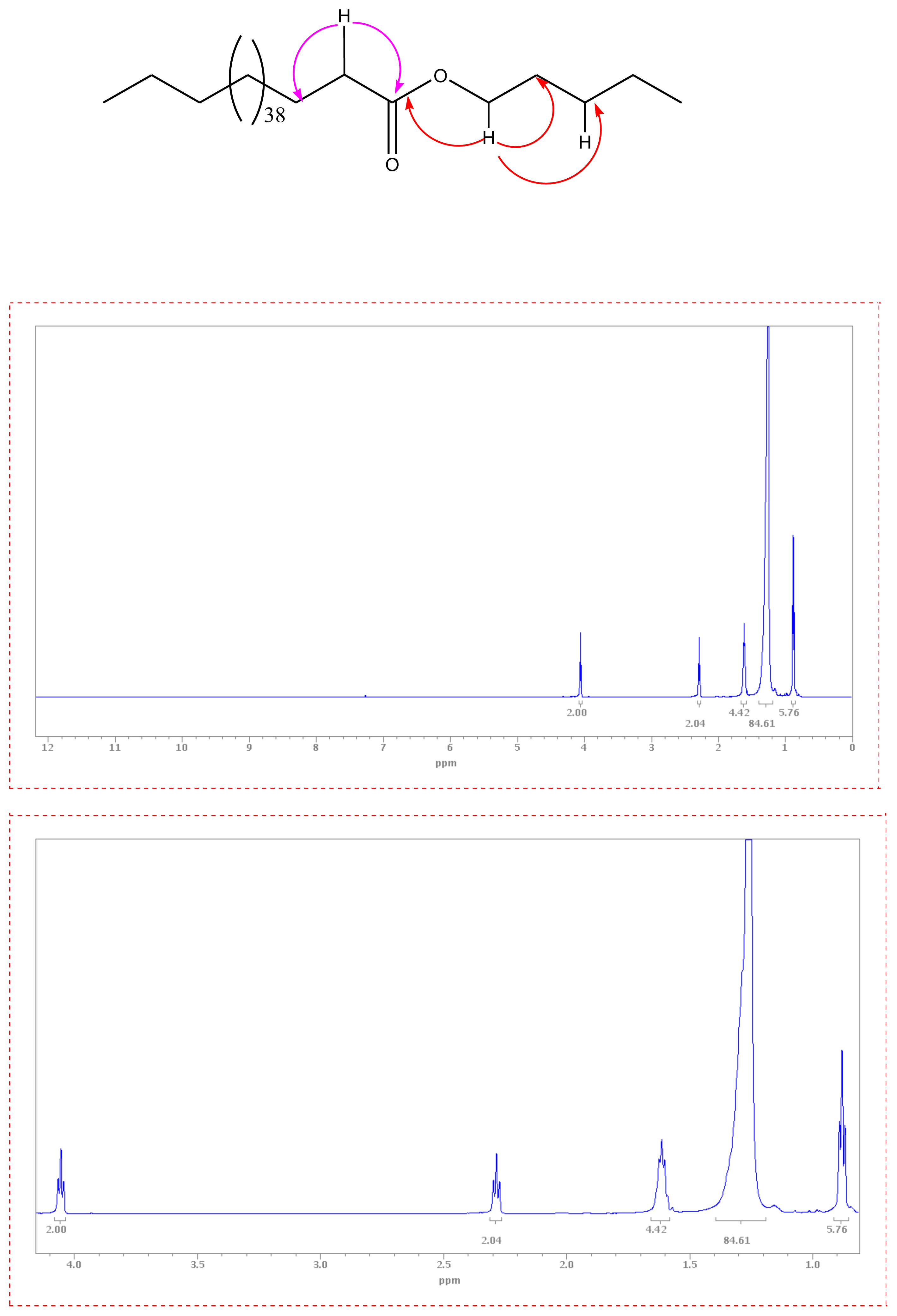

Supplement: Figure S1 — 1H NMR spectrum and HMBC correclations of compound 1 (600 MHz, CDCl3) [file turkjchem-46-1-169s1.tif]

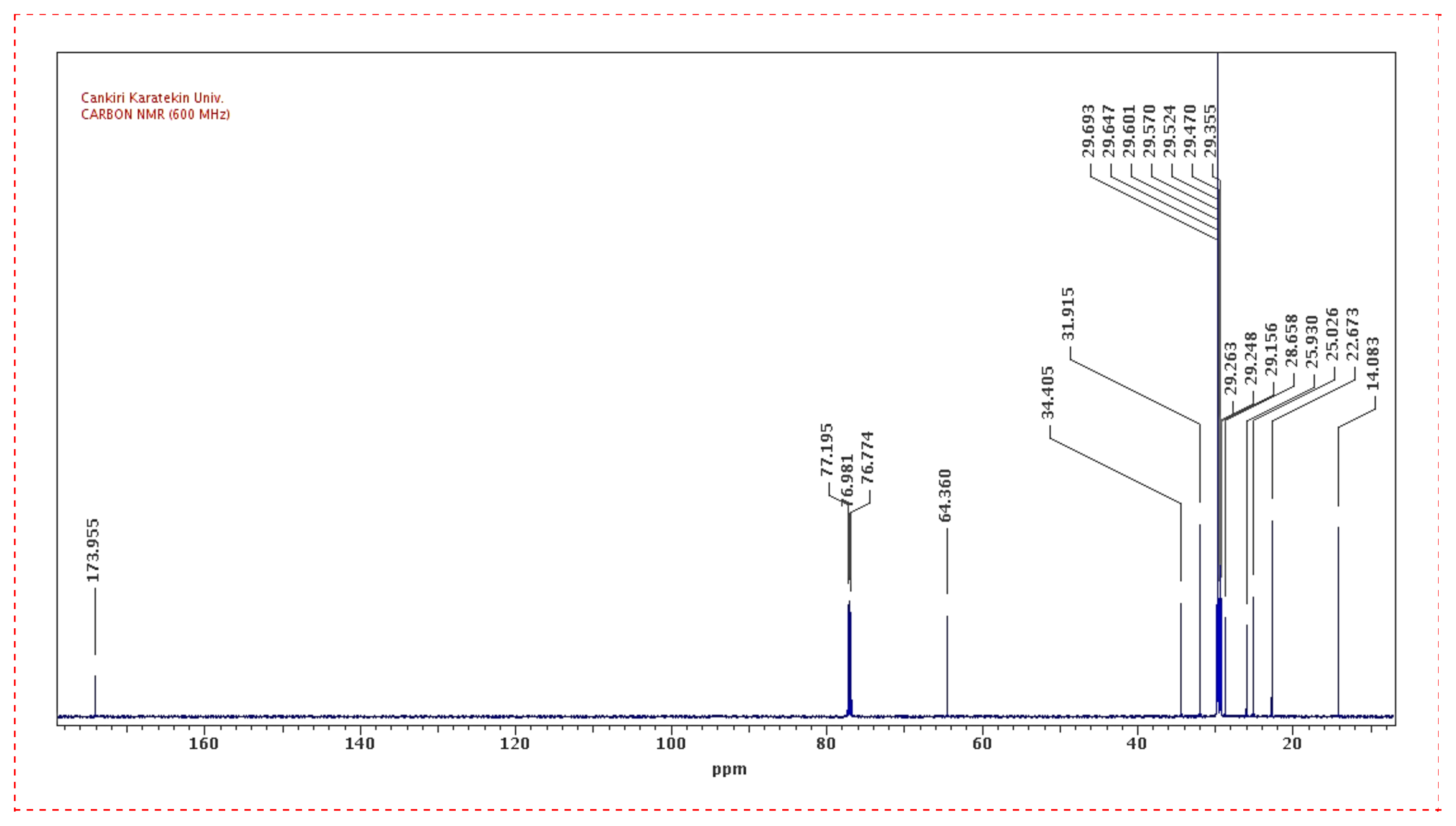

Supplement: Figure S2 — 13C NMR spectrum of compound 1 (150 MHz, CDCl3) [file turkjchem-46-1-169s2.tif]

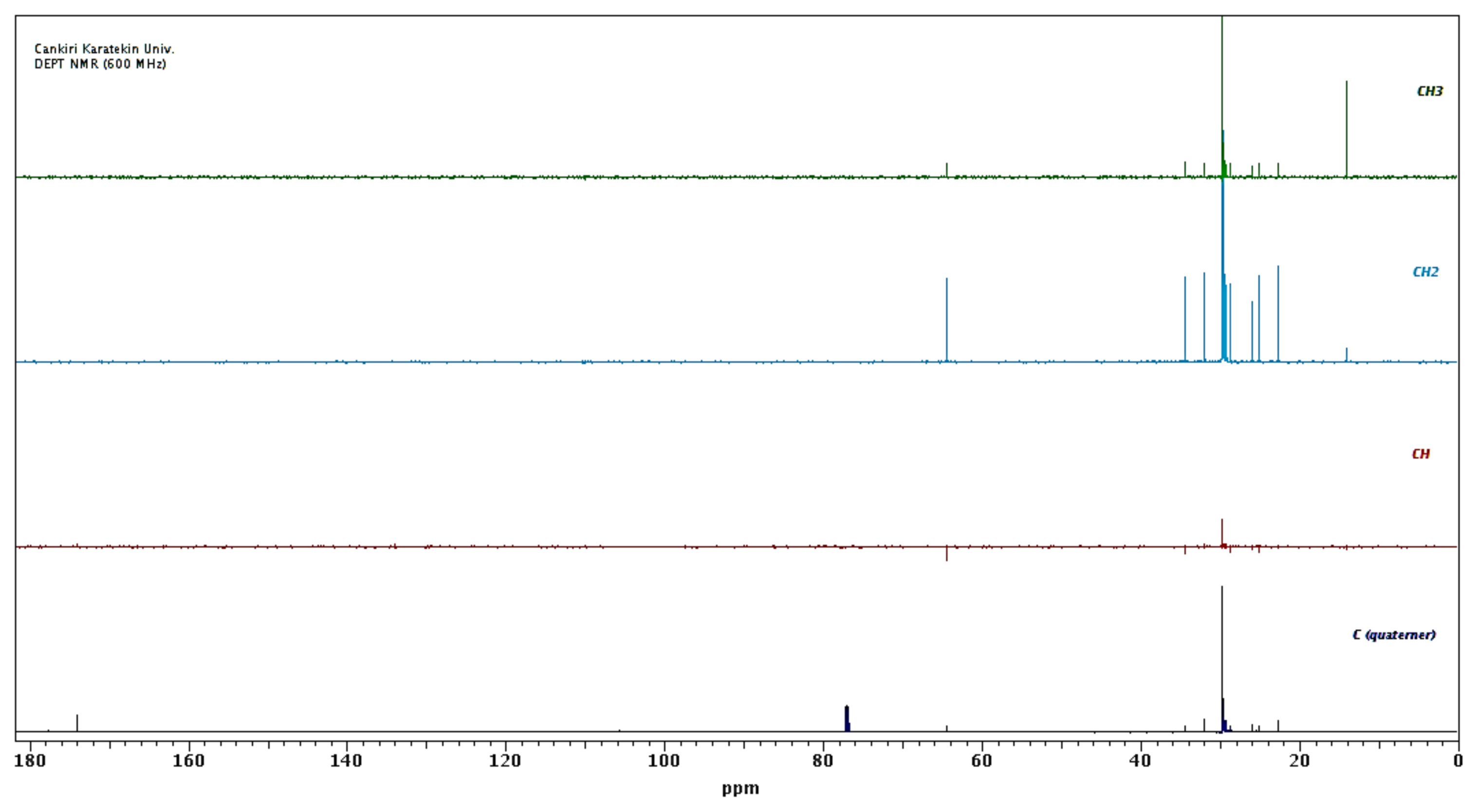

Supplement: Figure S3 — DEPT NMR spectrum of compound 1 (150 MHz, CDCl3) [file turkjchem-46-1-169s3.tif]

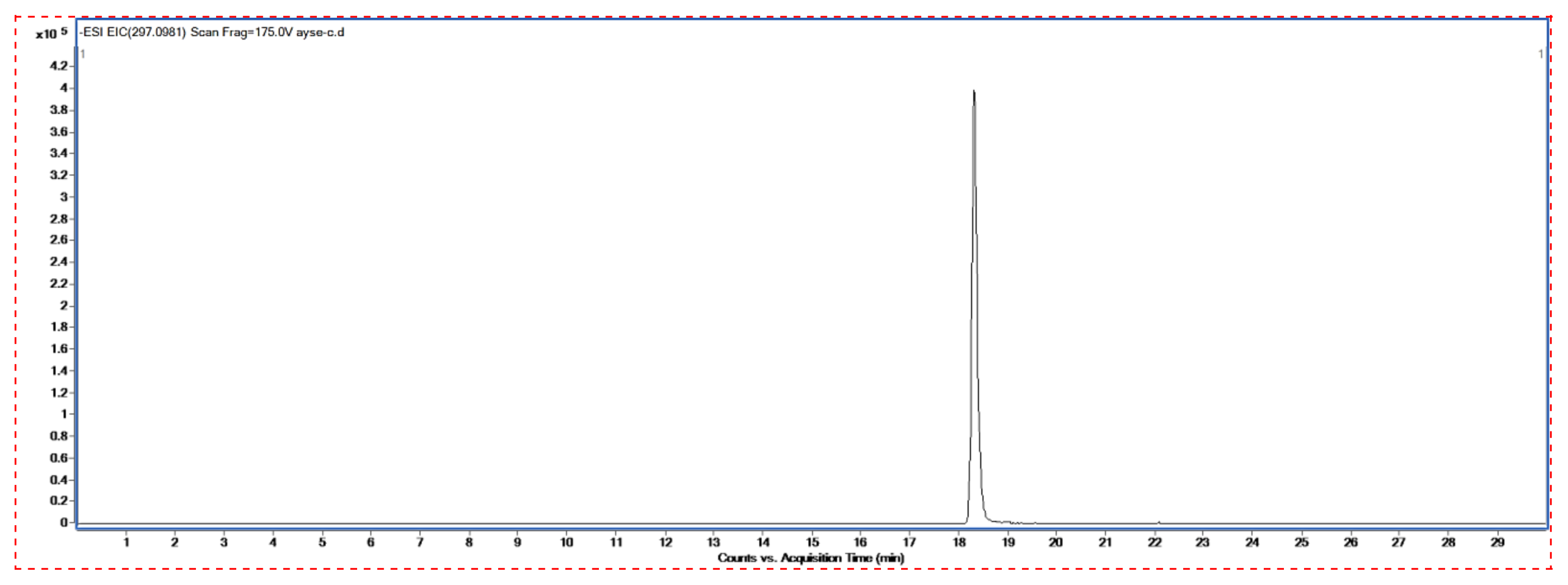

Supplement: Figure S4 — HPLC/TOF-MS chromatogram of compound 2 [file turkjchem-46-1-169s4.tif]

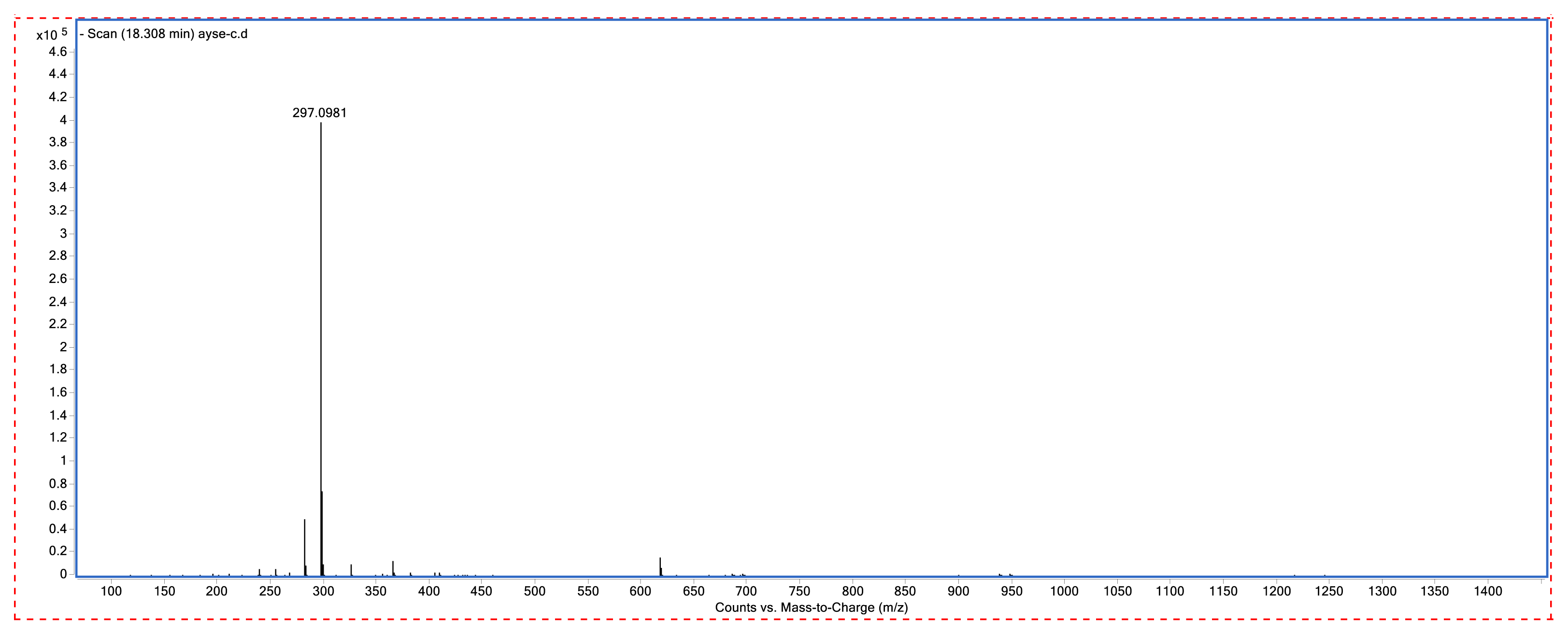

Supplement: Figure S5 — Mass spectrum of compound 2 [file turkjchem-46-1-169s5.tif]

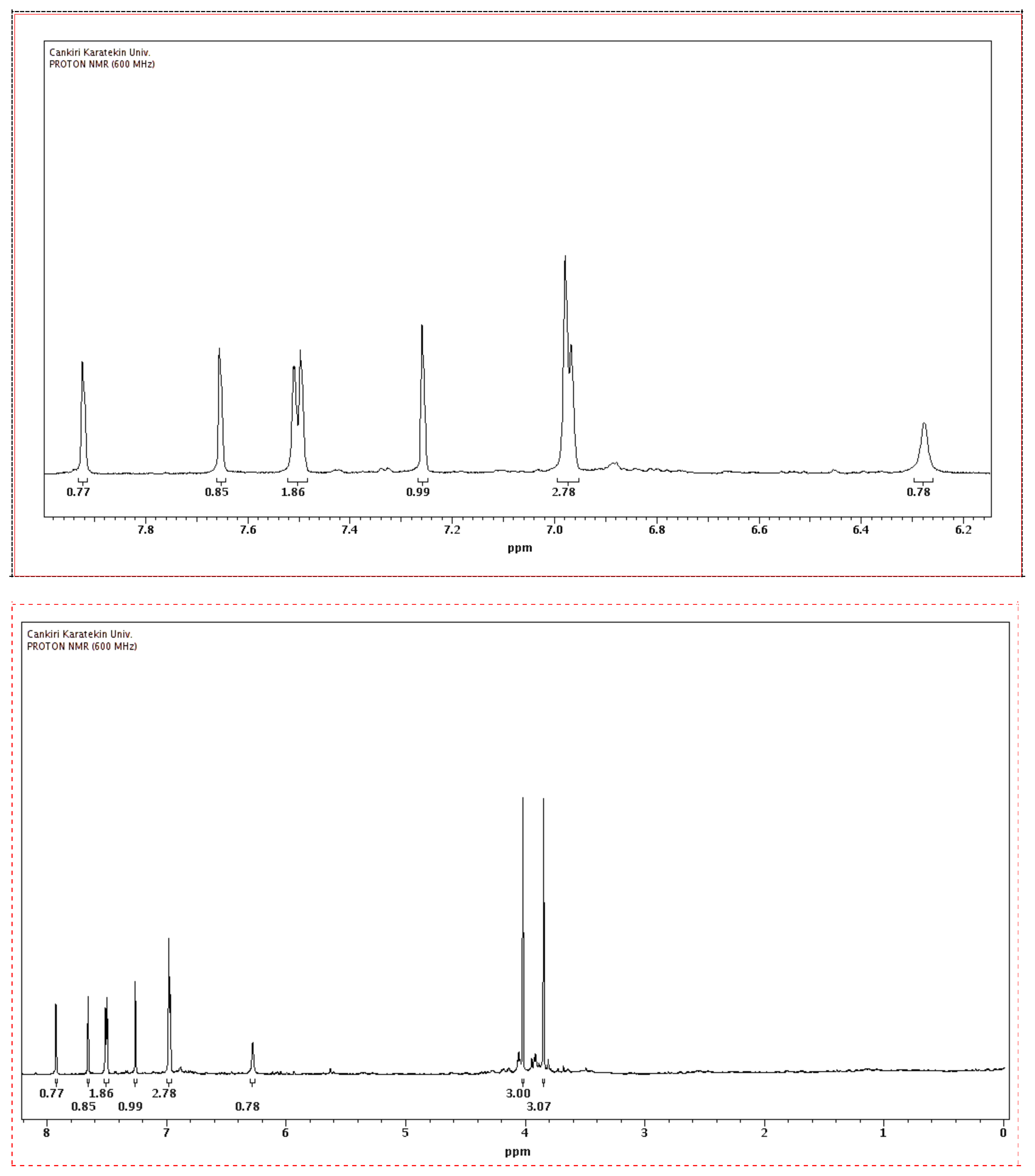

Supplement: Figure S6 — 1H NMR spectrum of compound 2 (600 MHz, CDCl3) [file turkjchem-46-1-169s6.tif]

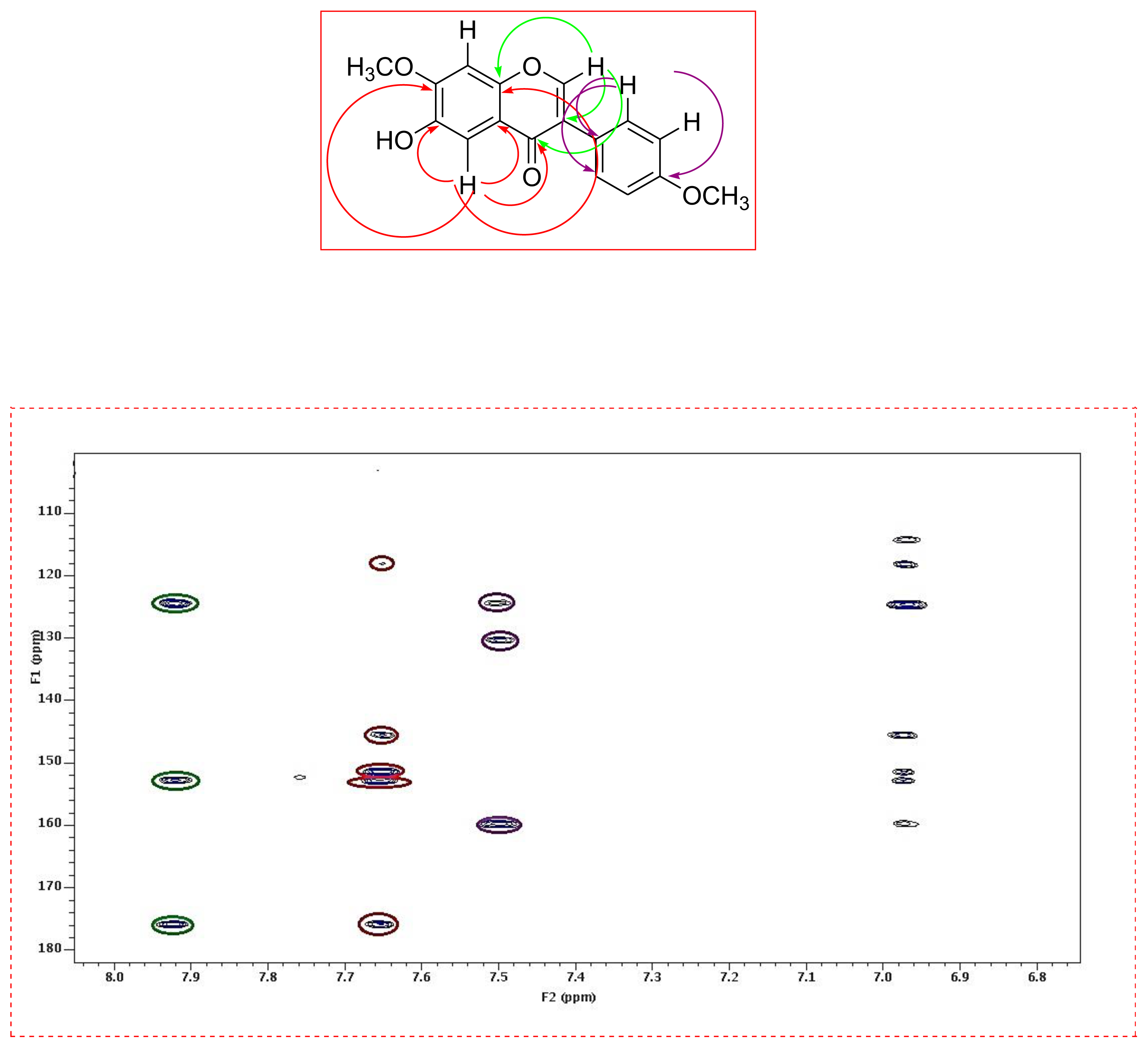

Supplement: Figure S7 — HMBC spectrum of compound 2 (600 MHz, CDCl3) [file turkjchem-46-1-169s7.tif]

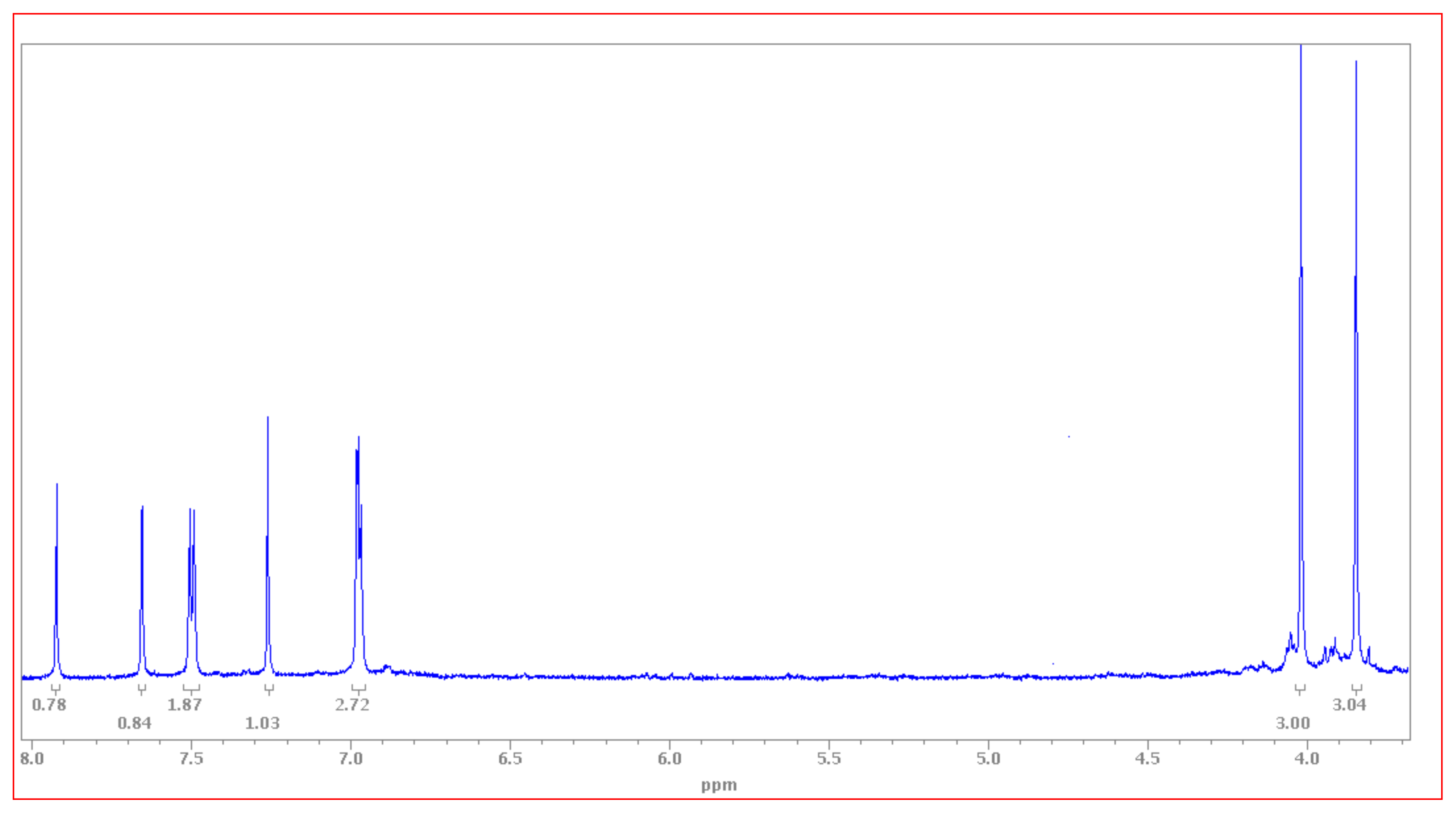

Supplement: Figure S8 — 1H NMR spectrum of compound 2 taken with D2O (600 MHz, CDCl3) [file turkjchem-46-1-169s8.tif]

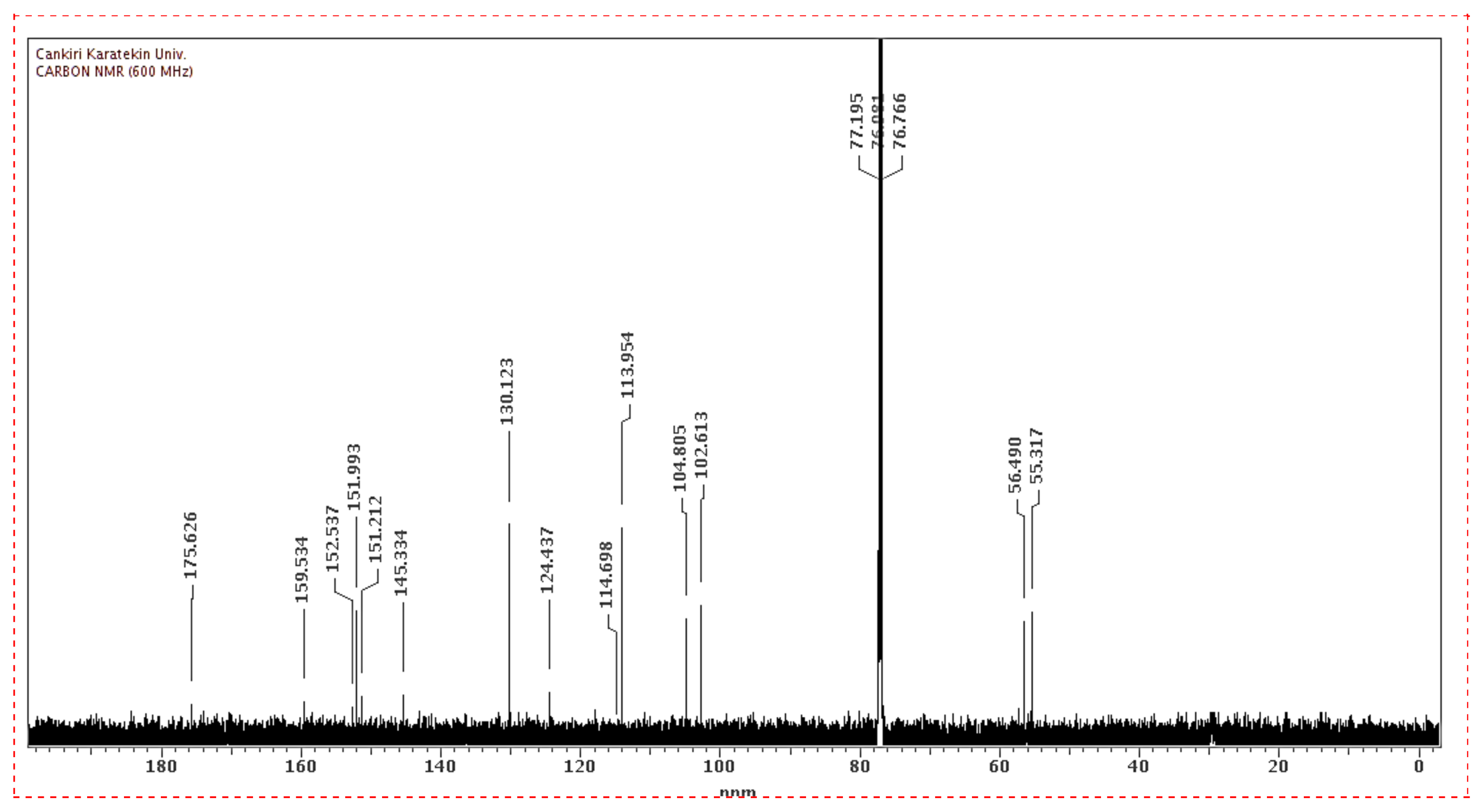

Supplement: Figure S9 — 13C NMR spectrum of compound 2 (150 MHz, CDCl3) [file turkjchem-46-1-169s9.tif]

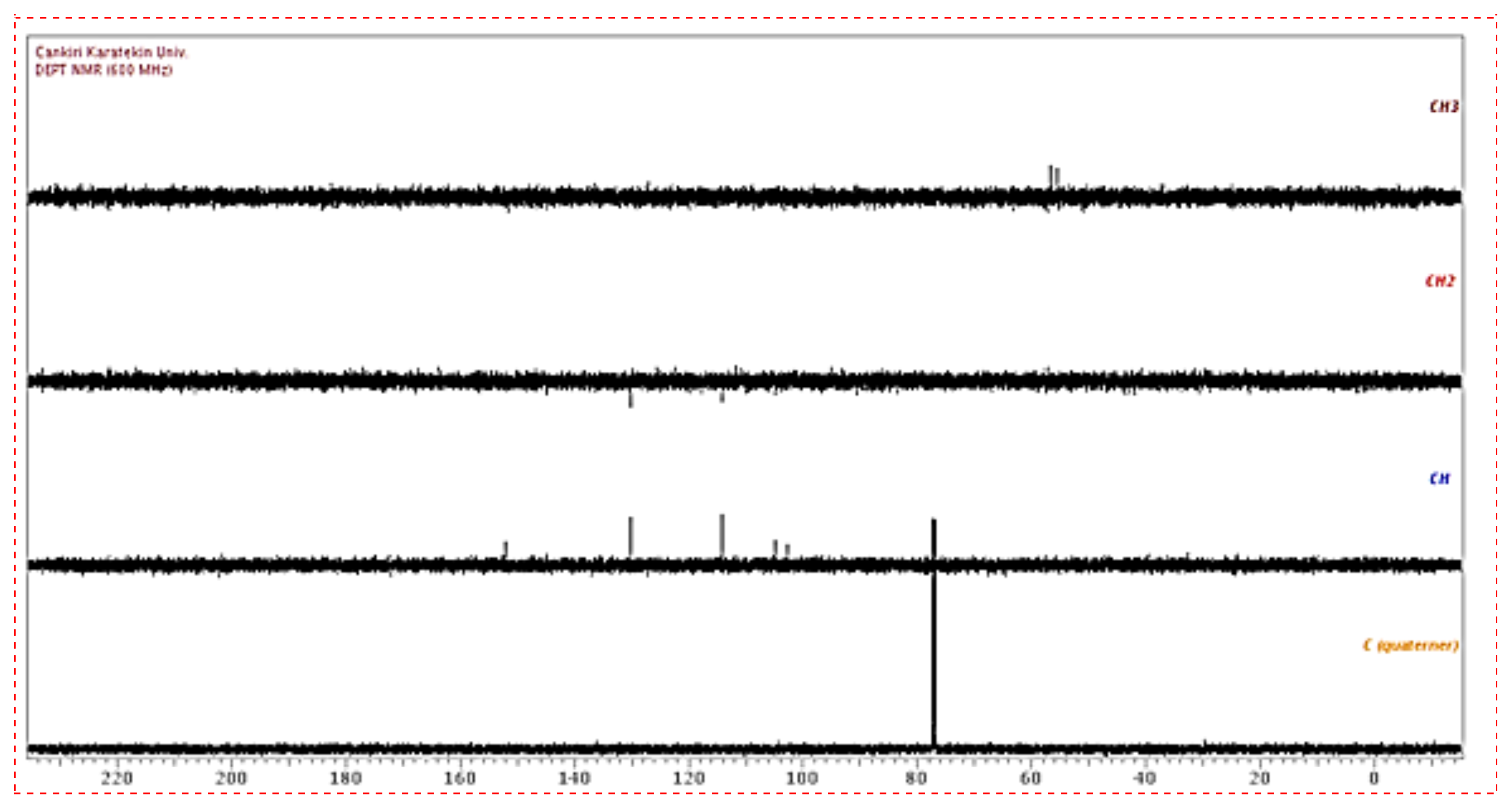

Supplement: Figure S10 — DEPT NMR spectrum of compound 2 (150 MHz, CDCl3) [file turkjchem-46-1-169s10.tif]

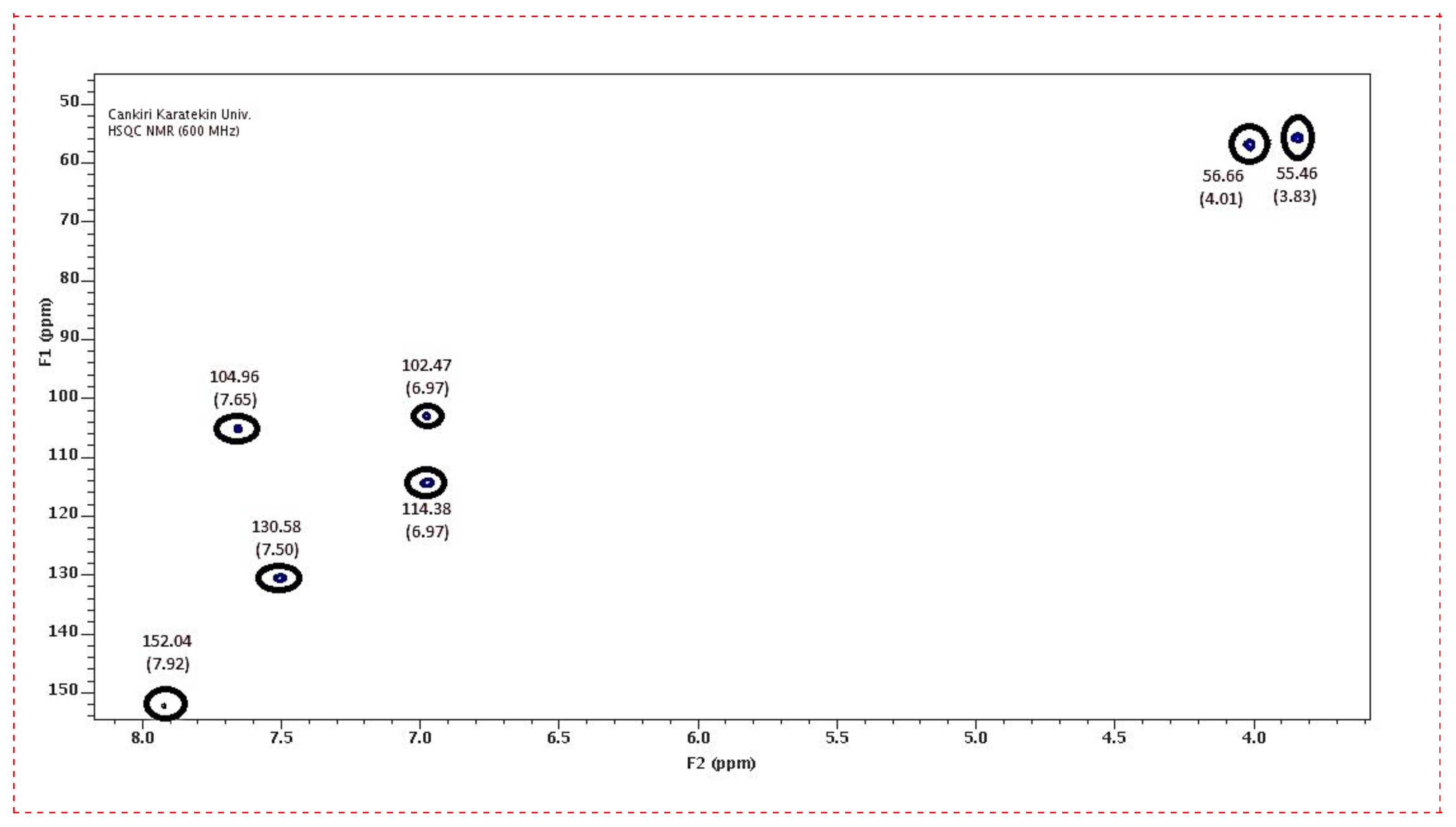

Supplement: Figure S11 — HSQC NMR spectrum of compound 2 (600 MHz, CDCl3) [file turkjchem-46-1-169s11.tif]

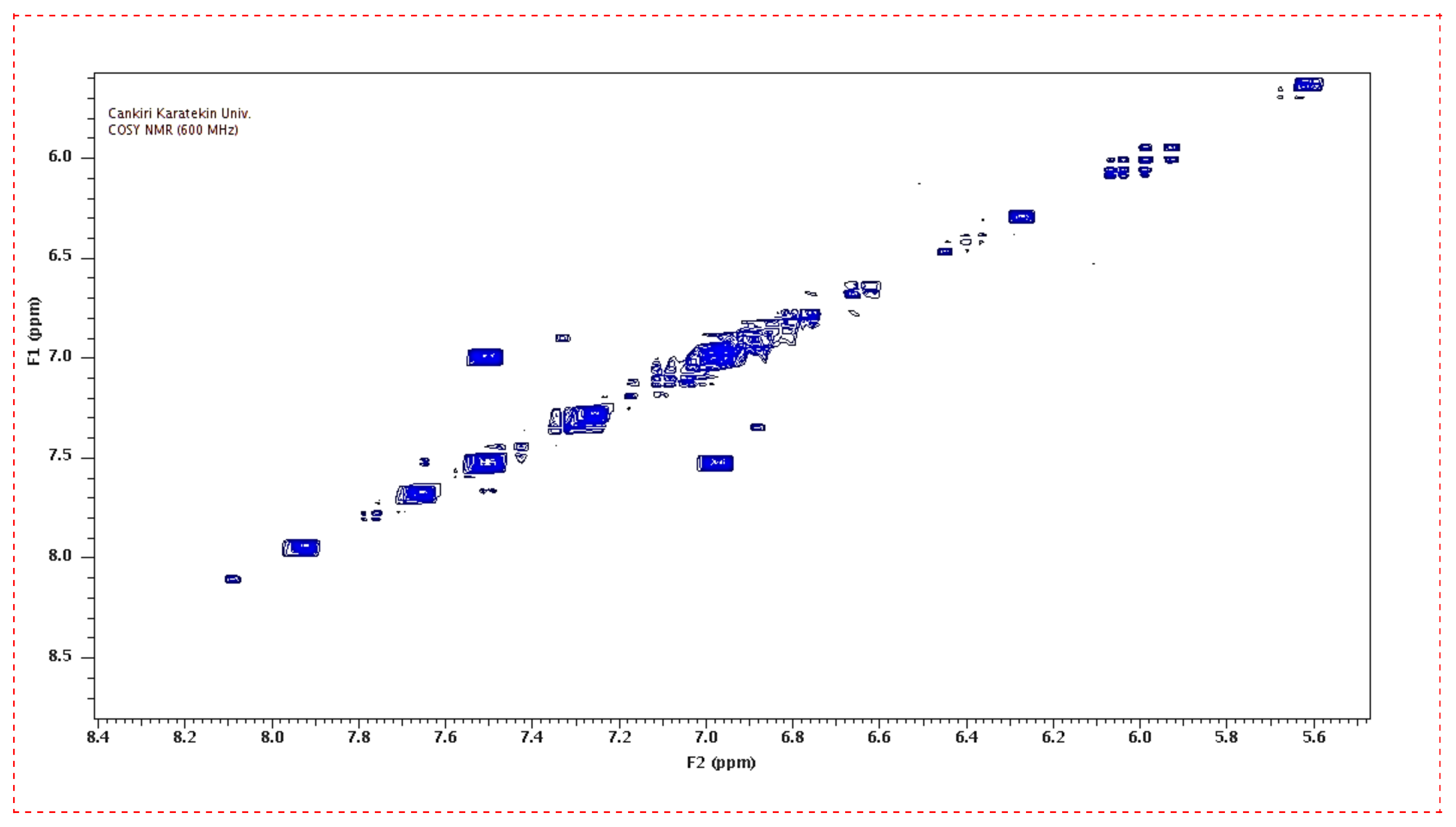

Supplement: Figure S12 — COSY NMR spectrum of compound 2 (600 MHz, CDCl3) [file turkjchem-46-1-169s12.tif]

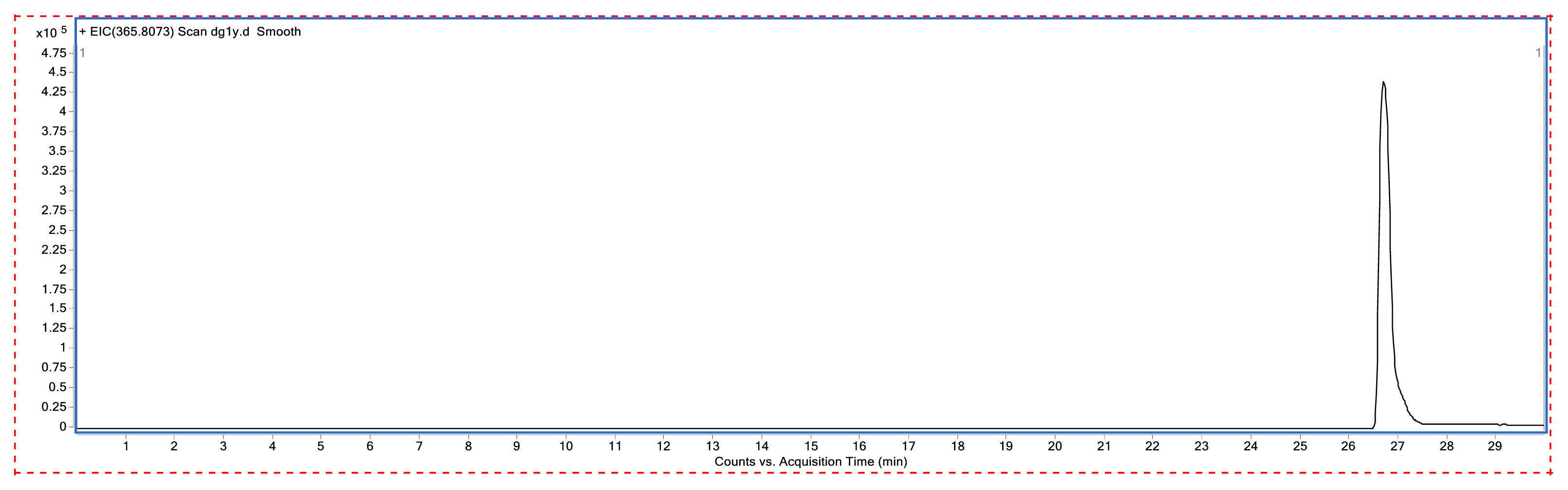

Supplement: Figure S13 — HPLC/TOF-MS chromatogram of compound 3–4 mix [file turkjchem-46-1-169s13.tif]

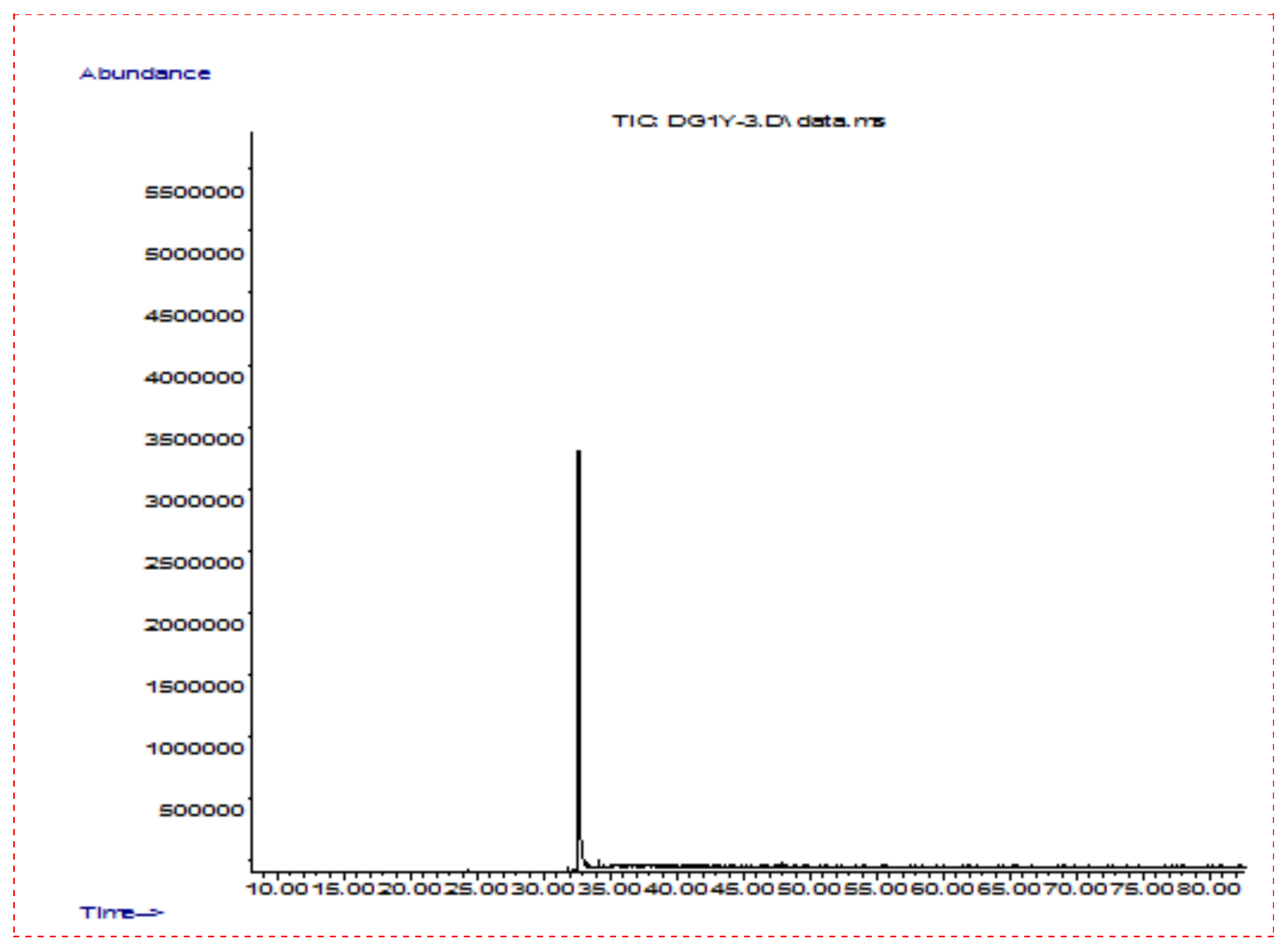

Supplement: Figure S14 — GC-MS chromatogram of compound 3–4 mix [file turkjchem-46-1-169s14.tif]

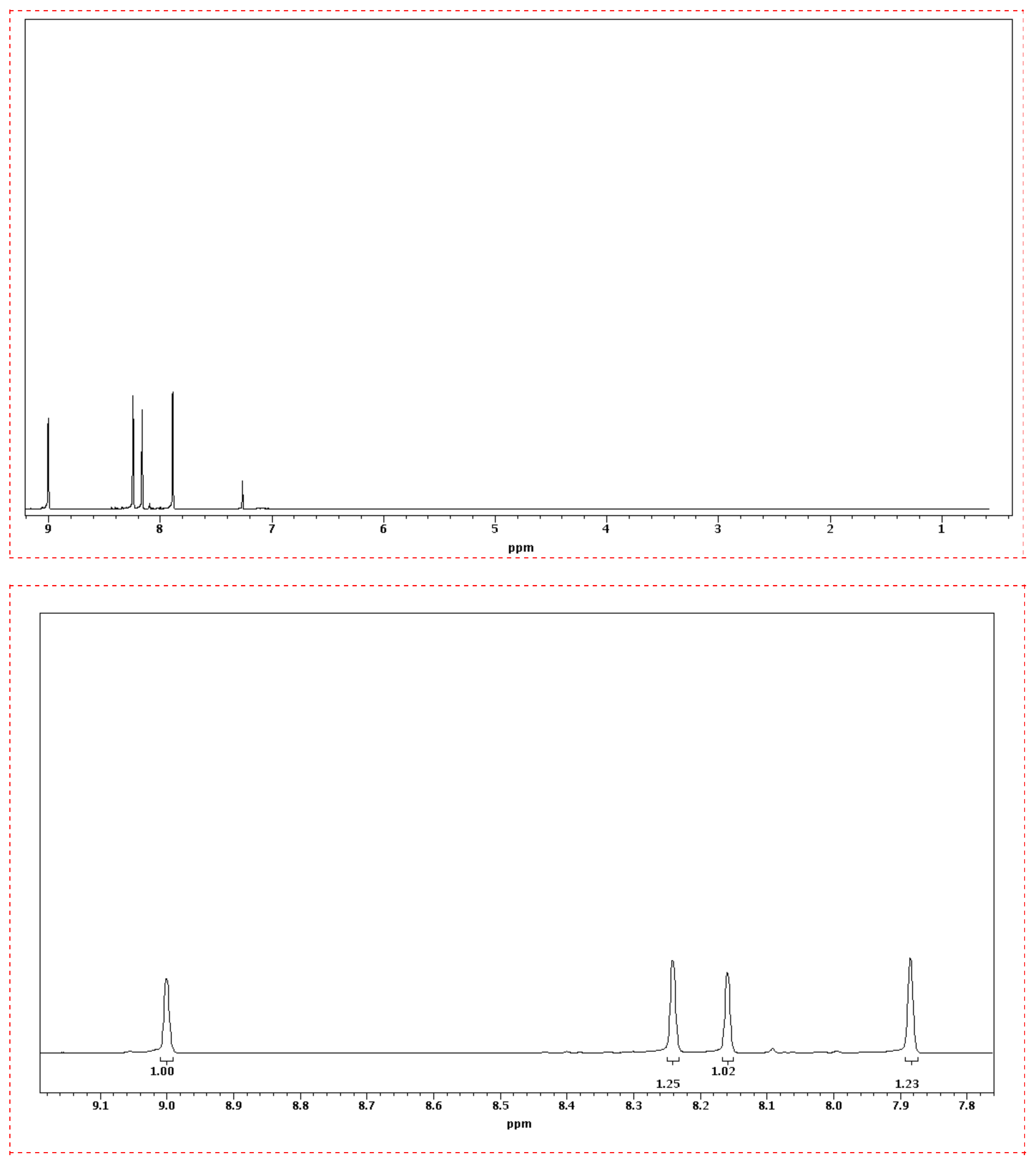

Supplement: Figure S15 — 1H NMR spectrum of compound 3–4 mix (600 MHz, CDCl3) [file turkjchem-46-1-169s15.tif]

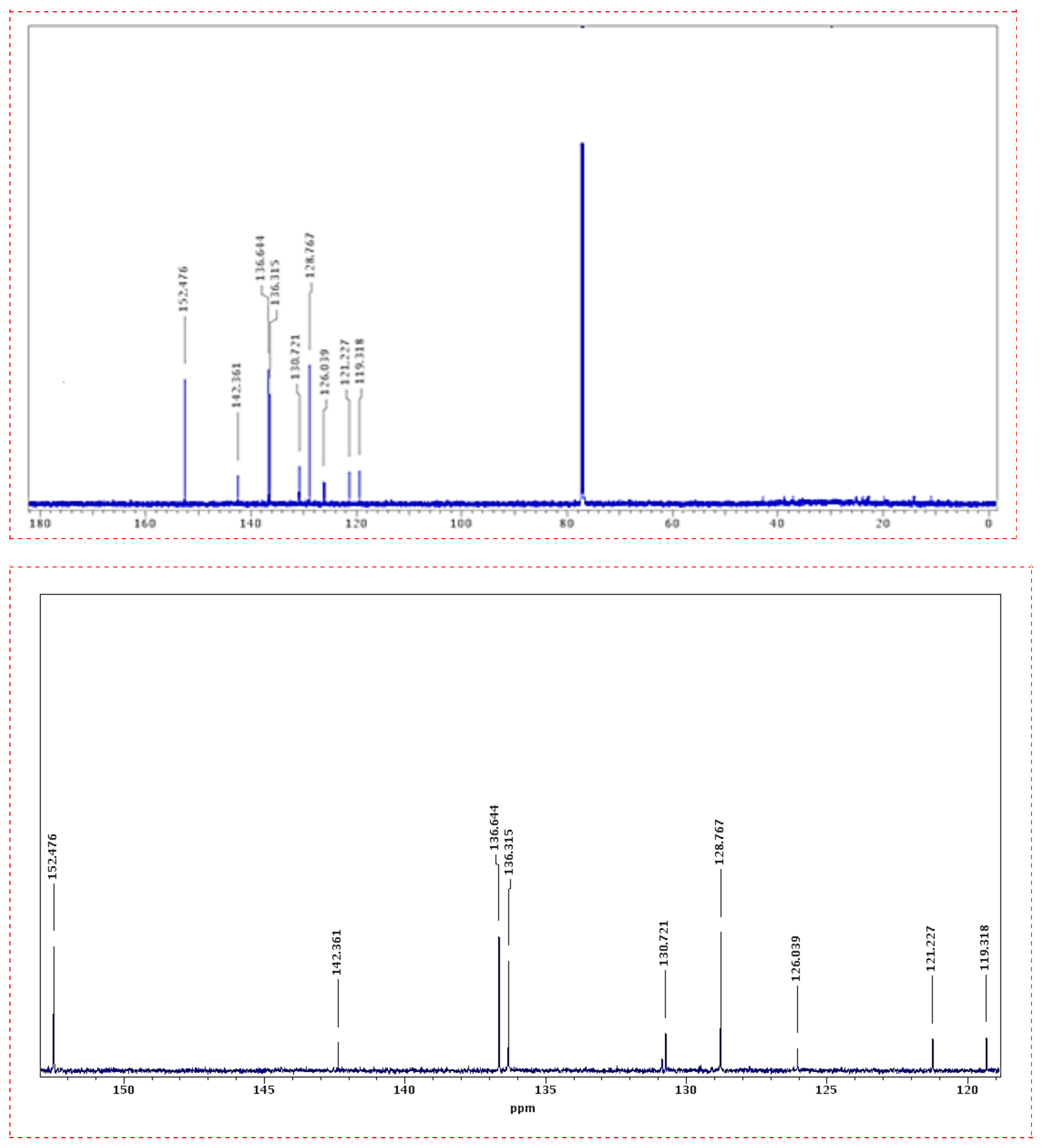

Supplement: Figure S16 — 13C NMR spectrum of compound 3–4 mix (150 MHz, CDCl3) [file turkjchem-46-1-169s16.tif]

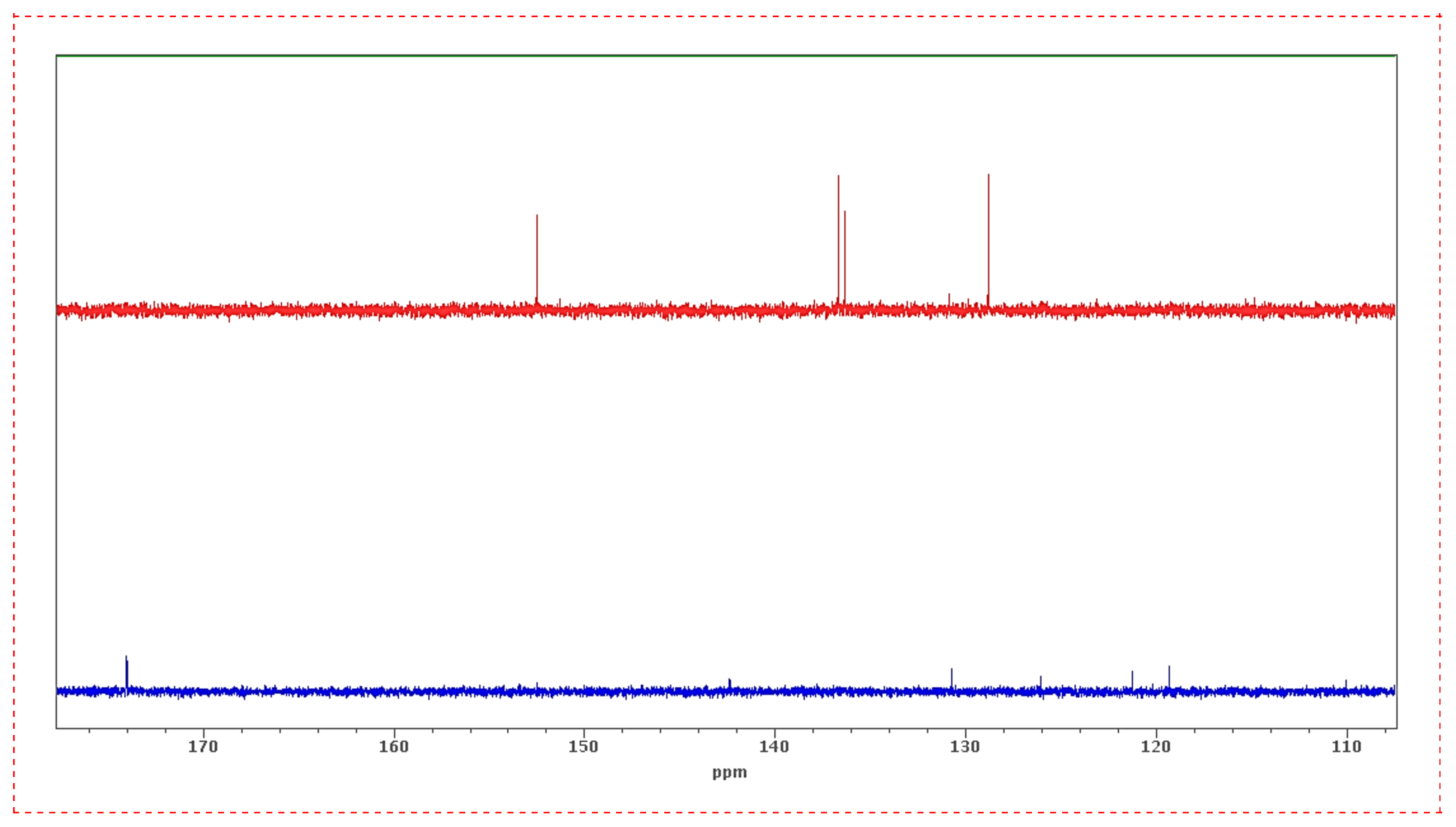

Supplement: Figure S17 — DEPT NMR spectrum of compound 3–4 mix (150 MHz, CDCl3) [file turkjchem-46-1-169s17.tif]

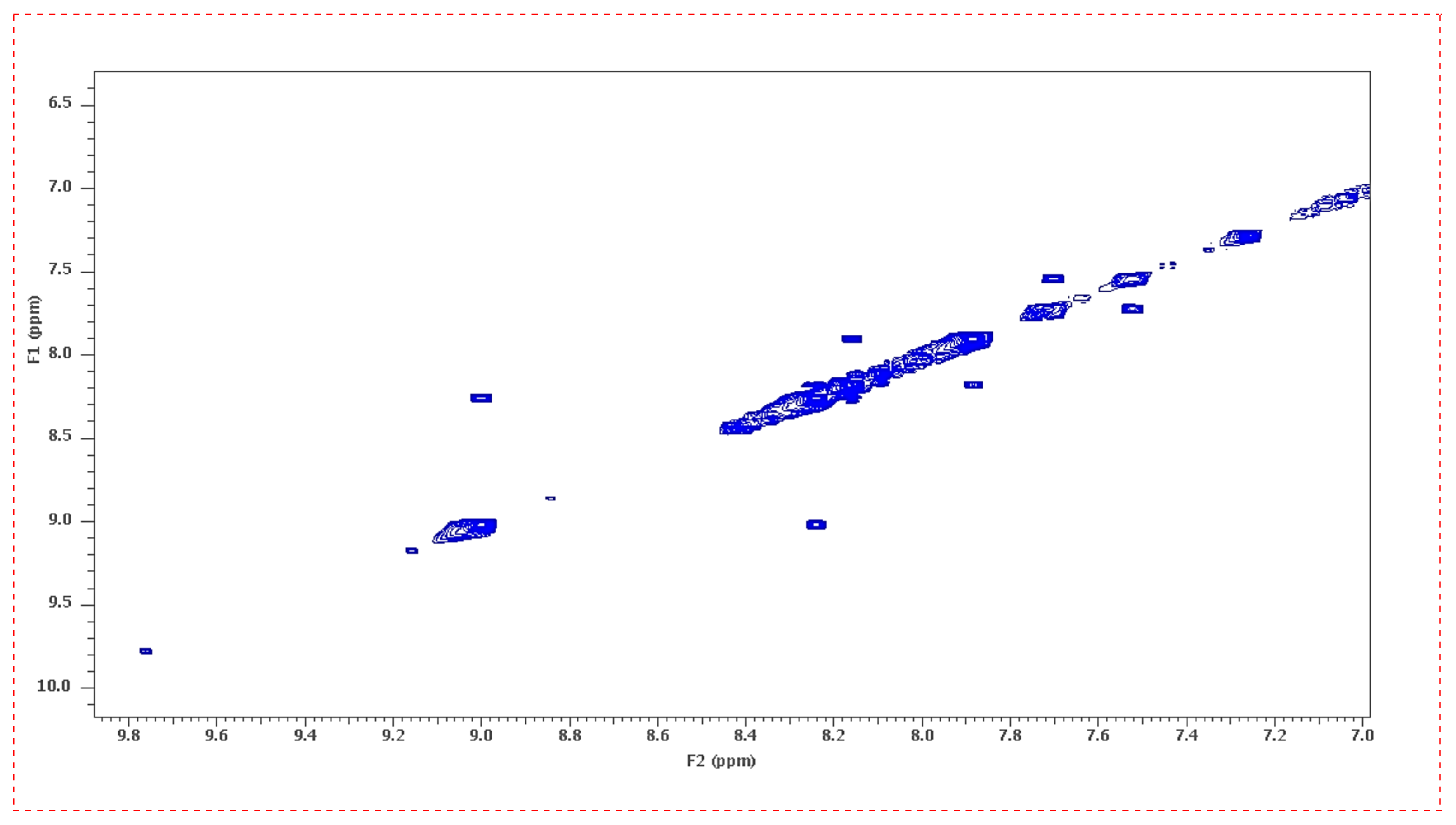

Supplement: Figure S18 — COSY spectrum of compound 3–4 mix (600 MHz, CDCl3) [file turkjchem-46-1-169s18.tif]

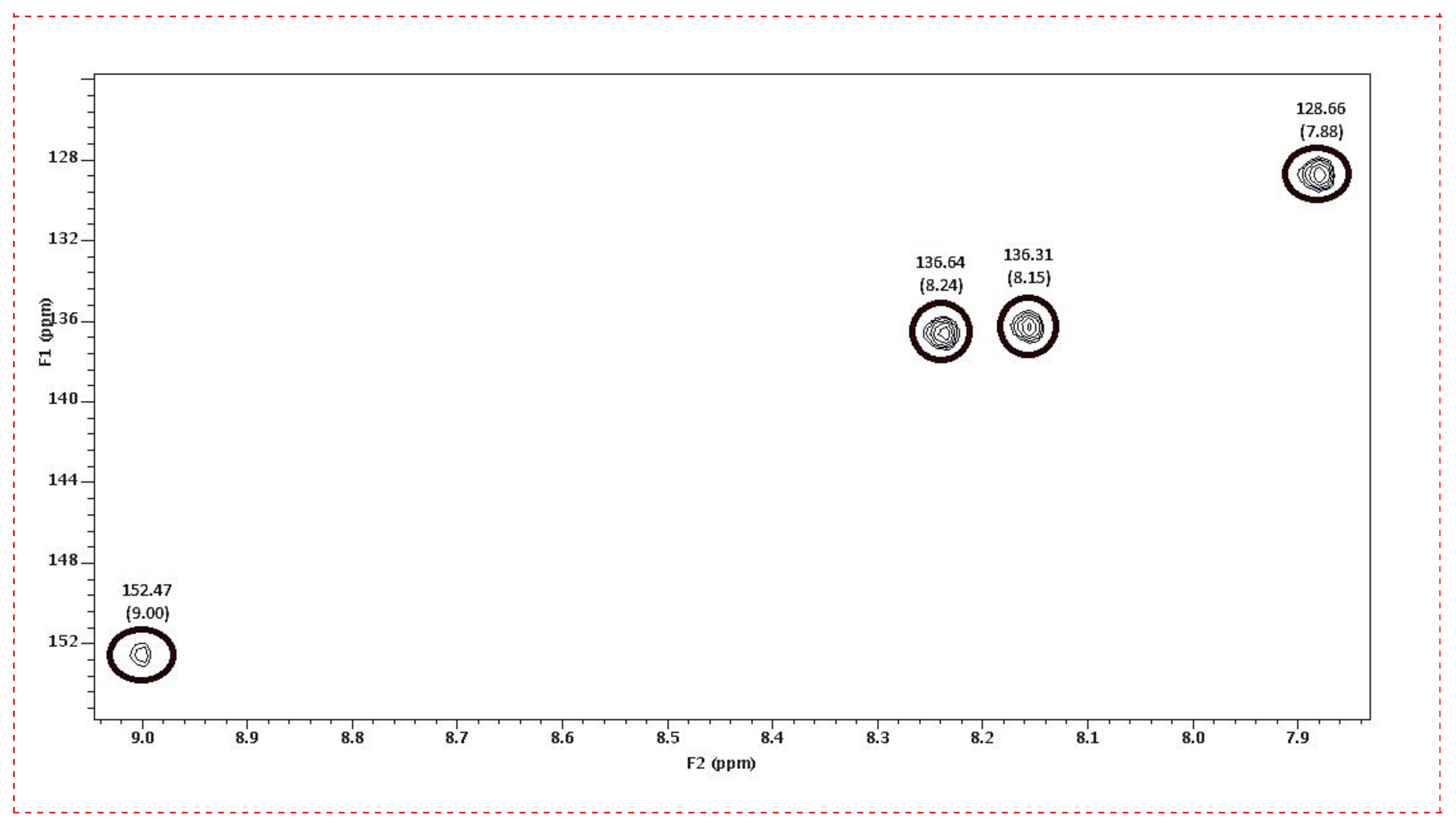

Supplement: Figure S19 — HSQC spectrum of compound 3–4 mix (600 MHz, CDCl3) [file turkjchem-46-1-169s19.tif]

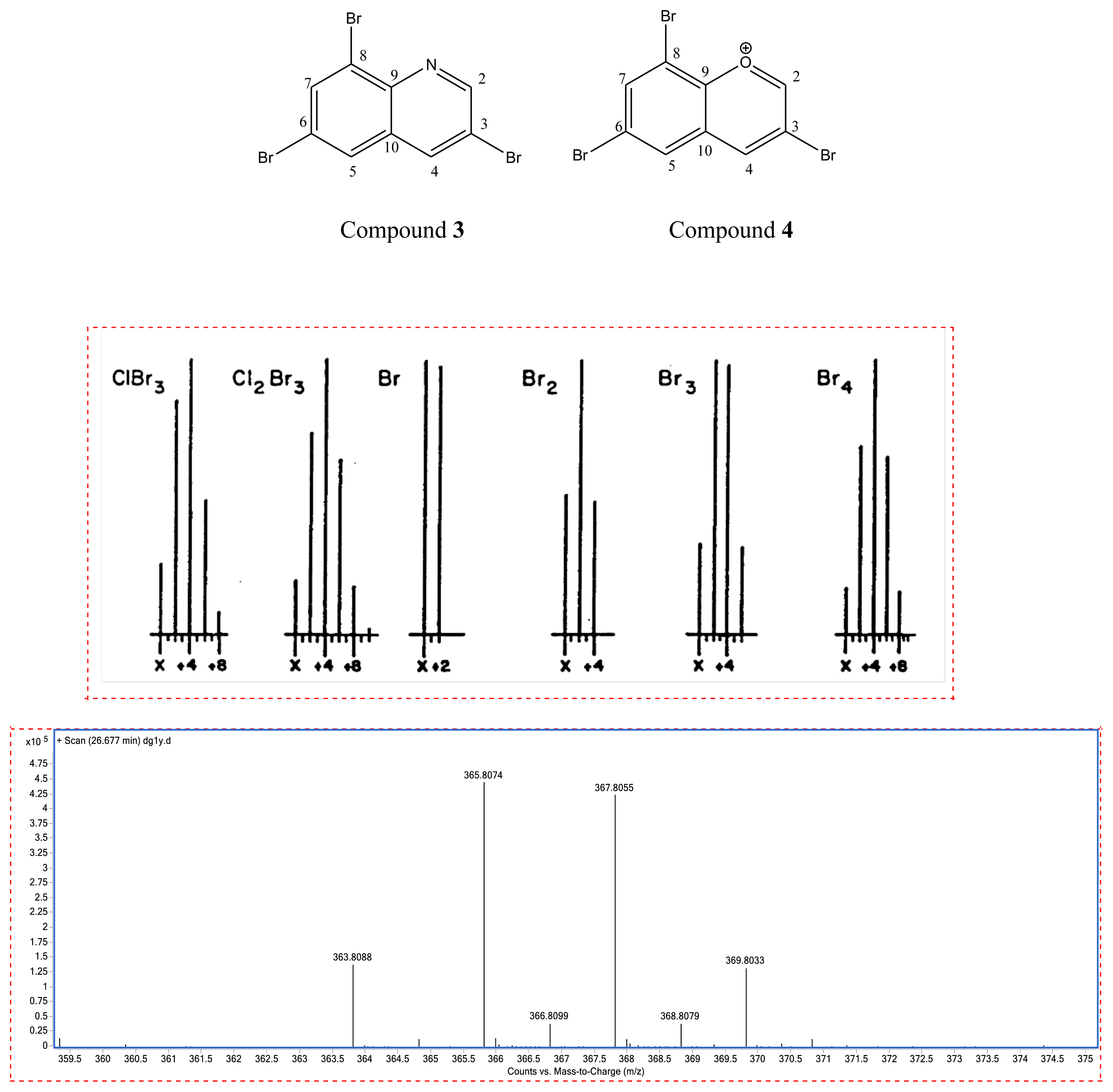

Supplement: Figure S20 — The mass spectra and structure of compound 3–4 mix [file turkjchem-46-1-169s20.tif]

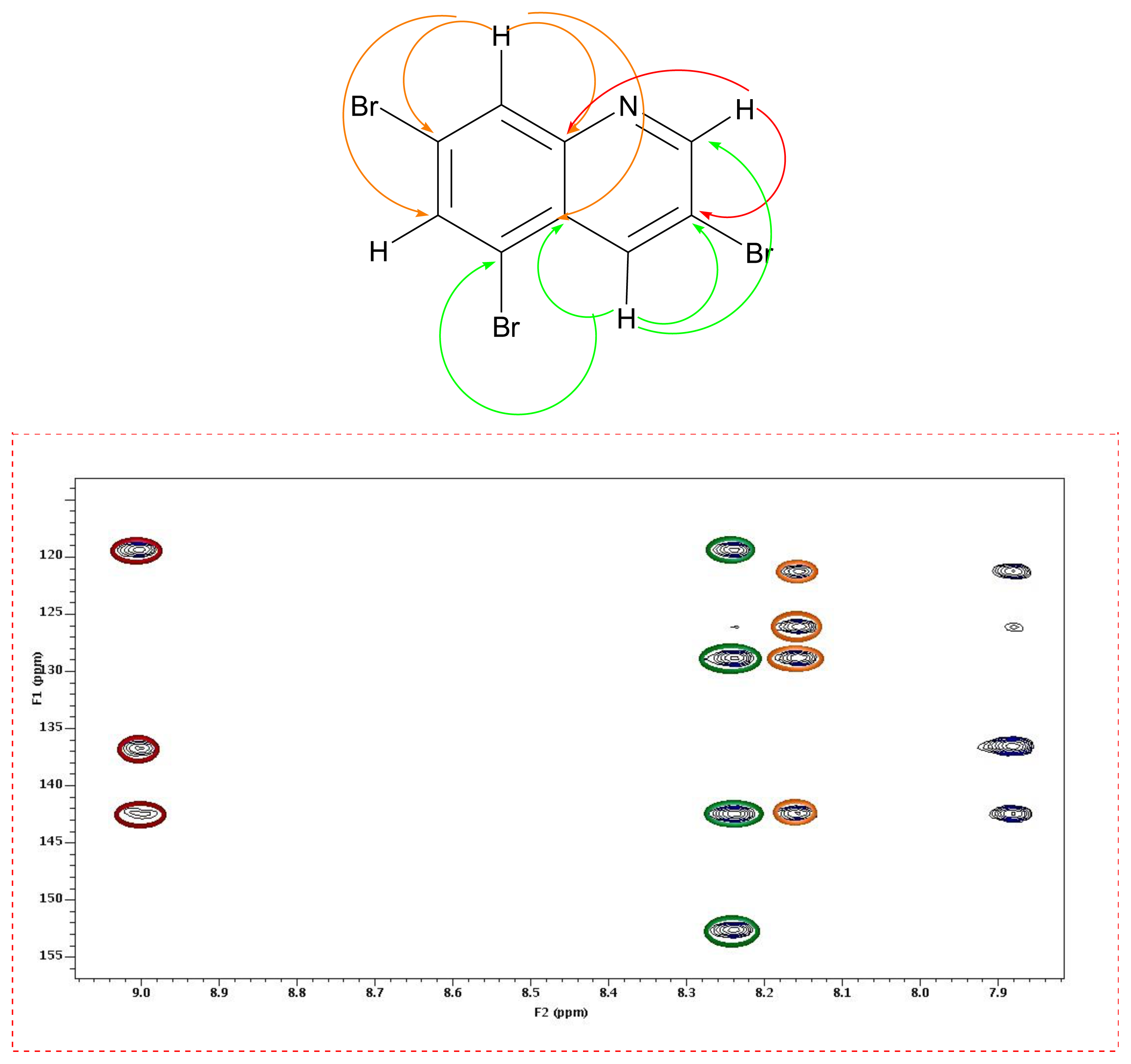

Supplement: Figure S21 — HMBC NMR spectrum and correlations of compound 3–4 mix (600 MHz, CDCl3) [file turkjchem-46-1-169s21.tif]
